# Amino acid 138 in the HA of a H3N2 subtype influenza A virus increases affinity for the lower respiratory tract and alveolar macrophages in pigs

**DOI:** 10.1371/journal.ppat.1012026

**Published:** 2024-02-20

**Authors:** Matias Cardenas, Brittany Seibert, Brianna Cowan, Ana Luiza S. Fraiha, Silvia Carnaccini, L. Claire Gay, Flavio Cargnin Faccin, C. Joaquin Caceres, Tavis K. Anderson, Amy L. Vincent Baker, Daniel R. Perez, Daniela S. Rajao

**Affiliations:** 1 Department of Population Health, College of Veterinary Medicine, University of Georgia, Athens, Georgia, United States of America; 2 Virus and Prion Research Unit, National Animal Disease Center, United States Department of Agriculture, Agricultural Research Service, Ames, Iowa, United States of America; University of Pittsburgh, UNITED STATES

## Abstract

Influenza A virus (FLUAV) infects a wide range of hosts and human-to-swine spillover events are frequently reported. However, only a few of these human viruses have become established in pigs and the host barriers and molecular mechanisms driving adaptation to the swine host remain poorly understood. We previously found that infection of pigs with a 2:6 reassortant virus (hVIC/11) containing the hemagglutinin (HA) and neuraminidase (NA) gene segments from the human strain A/Victoria/361/2011 (H3N2) and internal gene segments of an endemic swine strain (sOH/04) resulted in a fixed amino acid substitution in the HA (A138S, mature H3 HA numbering). In silico analysis revealed that S138 became predominant among swine H3N2 virus sequences deposited in public databases, while 138A predominates in human isolates. To understand the role of the HA A138S substitution in the adaptation of a human-origin FLUAV HA to swine, we infected pigs with the hVIC/11^A138S^ mutant and analyzed pathogenesis and transmission compared to hVIC/11 and sOH/04. Our results showed that the hVIC/11^A138S^ virus had an intermediary pathogenesis between hVIC/11 and sOH/04. The hVIC/11^A138S^ infected the upper respiratory tract, right caudal, and both cranial lobes while hVIC/11 was only detected in nose and trachea samples. Viruses induced a distinct expression pattern of various pro-inflammatory cytokines such as IL-8, TNF-α, and IFN-β. Flow cytometric analysis of lung samples revealed a significant reduction of porcine alveolar macrophages (PAMs) in hVIC/11^A138S^-infected pigs compared to hVIC/11 while a MHCII^low^CD163^neg^ population was increased. The hVIC/11^A138S^ showed a higher affinity for PAMs than hVIC/11, noted as an increase of infected PAMs in bronchoalveolar lavage fluid (BALF), and showed no differences in the percentage of HA-positive PAMs compared to sOH/04. This increased infection of PAMs led to an increase of granulocyte-monocyte colony-stimulating factor (GM-CSF) stimulation but a reduced expression of peroxisome proliferator-activated receptor gamma (PPARγ) in the sOH/04-infected group. Analysis using the PAM cell line 3D4/21 revealed that the A138S substitution improved replication and apoptosis induction in this cell type compared to hVIC/11 but at lower levels than sOH/04. Overall, our study indicates that adaptation of human viruses to the swine host involves an increased affinity for the lower respiratory tract and alveolar macrophages.

## Author summary

Human-to-swine FLUAV spillover events are relatively common and have led to the establishment of multiple evolutionarily distinct virus lineages in the swine population worldwide, leading to important economic losses in the pork industry. This also increases the risk of reassortment and emergence of novel FLUAV that may retain the ability to infect and transmit in humans, an event exemplified by the H1N1 pandemic in 2009. However, the molecular mechanisms driving FLUAV adaptation to pigs following the introduction of a human virus are still not fully understood. In this study we found that a point mutation in a human-origin HA improved infection and transmissibility in pigs characterized by an enhanced lung replication and the disruption of lung-resident immune cell populations. This relationship between improved replication and infectivity of lung-resident cells provides insights into host barriers limiting human FLUAV adaptation to pigs and the complexities associated with the host immune response that influenza A viruses must overcome to become established in the swine population.

## Introduction

Influenza A viruses infect various animals, including birds, pigs, and humans [1]. While some strains have a restricted host range, it is well-documented that some viruses can jump between species [2]. A clear example is the emergence of the 2009 swine-origin H1N1 pandemic virus that rapidly spread worldwide in humans [3]. However, human-to-swine transmissions of FLUAV are more frequent than zoonotic events [4,5]. Although human-origin H1N1 and H3N2 FLUAV infections in pigs are frequent, H3N2 infections are generally self-limiting, and a reduced number of viruses evolve enough to become prevalent in the swine population [6]. One of the most recent examples is the spillover of a human-origin H3N2 during the 2010/2011 season that became established as a new H3N2 swine FLUAV lineage in North America (known as the 2010.1 lineage) [7]. Despite many of these human-origin viruses becoming established in swine and contributing to the diversity of viruses circulating in pigs globally, little is known about the biological processes driving human-to-swine adaptation. Previous reports showed that acquiring swine-origin FLUAV internal genes is critical [7,8]. Since the temperature of the lower respiratory tract of pigs is slightly higher than that of humans [5], the polymerase complex must be adapted to higher temperatures to overcome this host barrier, potentially through reassortment or gain of adaptative mutations [9,10]. It has also been shown that the HA gene can adapt to the new host via introduction of changes increasing receptor-binding affinity of the HA protein [11,12]. As a consequence, alterations of the NA gene are essential to maintain the balance between HA avidity and NA activity [13]. In addition, the ability of the virus to suppress or evade host-specific immune responses can also drive FLUAV evolution aiding in the establishment of viral infections during cross-species transmission.

Alveolar macrophages (AMs) are the most abundant immune cells in the lungs and account for up to 98% of cells in bronchoalveolar lavage fluid in pigs [14]. AMs contribute to the first line of defense against respiratory pathogens and are essential in developing innate and adaptative immune responses during FLUAV infection. Previous reports showed that they play a critical role in controlling FLUAV infection via the production of type I interferons and other pro- and anti-inflammatory cytokines such as TNF-α, IL-6, and IL-10 together with their intrinsic phagocytic activity [15,16]. Moreover, AMs play a significant role in building antibody-mediated protection against FLUAV, inducing and regulating the primary anti-FLUAV cytotoxic T-cell response [17,18]. AM-depleted animals infected with FLUAV have higher lung replication, overexpression of pro-inflammatory cytokines, increased tissue damage and higher mortality, underscoring that AMs are imperative for combatting FLUAV infection [19–21]. While AMs are essential for the anti-influenza immune response, they have been previously reported to be susceptible to FLUAV infection [22,23]. However, numerous FLUAV HA subtypes show limited virus replication within these cells except for a subset of both highly pathogenic and low pathogenic H5 viruses that efficiently replicate in swine, mice, and human AMs [23–26]. Nonetheless, both H3 and H1 viruses have also been described to infect mice AMs [27]. Previous literature suggests that FLUAV infection of AMs not only induces death by apoptosis [21], but also impairs the immune activity of AMs via peroxisome proliferator-activated receptor gamma (PPARγ) repression [28,29]. This transcription factor regulates AMs activity and is activated after granulocyte-monocyte colony-stimulating factor (GM-CSF) stimulation [30,31]. GM-CSF and PPARγ have also been described as main factors promoting monocyte differentiation into AMs and their proliferation [32,33]. However, the effect, if any, of FLUAV infection of AMs on virus tropism and host range is still unknown [34].

To assess the adaptation of human-derived HA to pigs and its implications on the swine immune response, we generated an H3N2 reassortant virus (hVIC/11) containing the HA and NA segments of a human seasonal A/Victoria/361/2011 (H3N2) virus and the remaining genes forming an internal gene constellation highly adapted to pigs. This internal gene constellation is formed by a combination of the triple reassortant internal gene (TRIG) cassette and the 2009 pandemic matrix (M) gene which was the most prevalent constellation circulating from 2014–2019 in North American swine herds. When pigs were inoculated with the hVIC/11 virus, a point mutation near the receptor-binding site of the HA protein (A138S) became fixed in contact pigs [35]. This mutation improved binding and replication in swine tracheal cells *in vitro* [35]. To further understand the impact of this mutation on pathogenesis and transmission, pigs were inoculated with a hVIC/11 virus carrying the A138S amino acid change (hVIC/11^A138S^) and compared to the original hVIC/11 and a swine-origin H3N2 virus A/turkey/Ohio/313053/2004 (sOH/04). We found that hVIC/11^A138S^ infected the upper and lower respiratory tract, while hVIC/11 was only detected in the upper respiratory tract by 5 days post infection (dpi). PAMs in bronchoalveolar lavage (BALF) samples were significantly decreased in sOH/04- and hVIC/11^A138S^-infected pigs but not in hVIC/11-infected animals. This reduction of PAMs was accompanied by an increased number of FLUAV-infected PAMs; however, only sOH/04 suppressed PPARγ expression. Upon further analysis using the porcine alveolar macrophage cell line 3D4/21, we found that the A138S mutation increases the virus’ ability to replicate and induce apoptosis in PAMs compared to hVIC/11 but at lower levels than sOH/04, suggesting that the decreased number of PAMs observed *in vivo* could be due to FLUAV-induced apoptosis.

## Results

### The A138S mutation modulates HA thermal stability, receptor-binding properties of hVIC/11^A138S^, and NA activity

**Fig 1:**
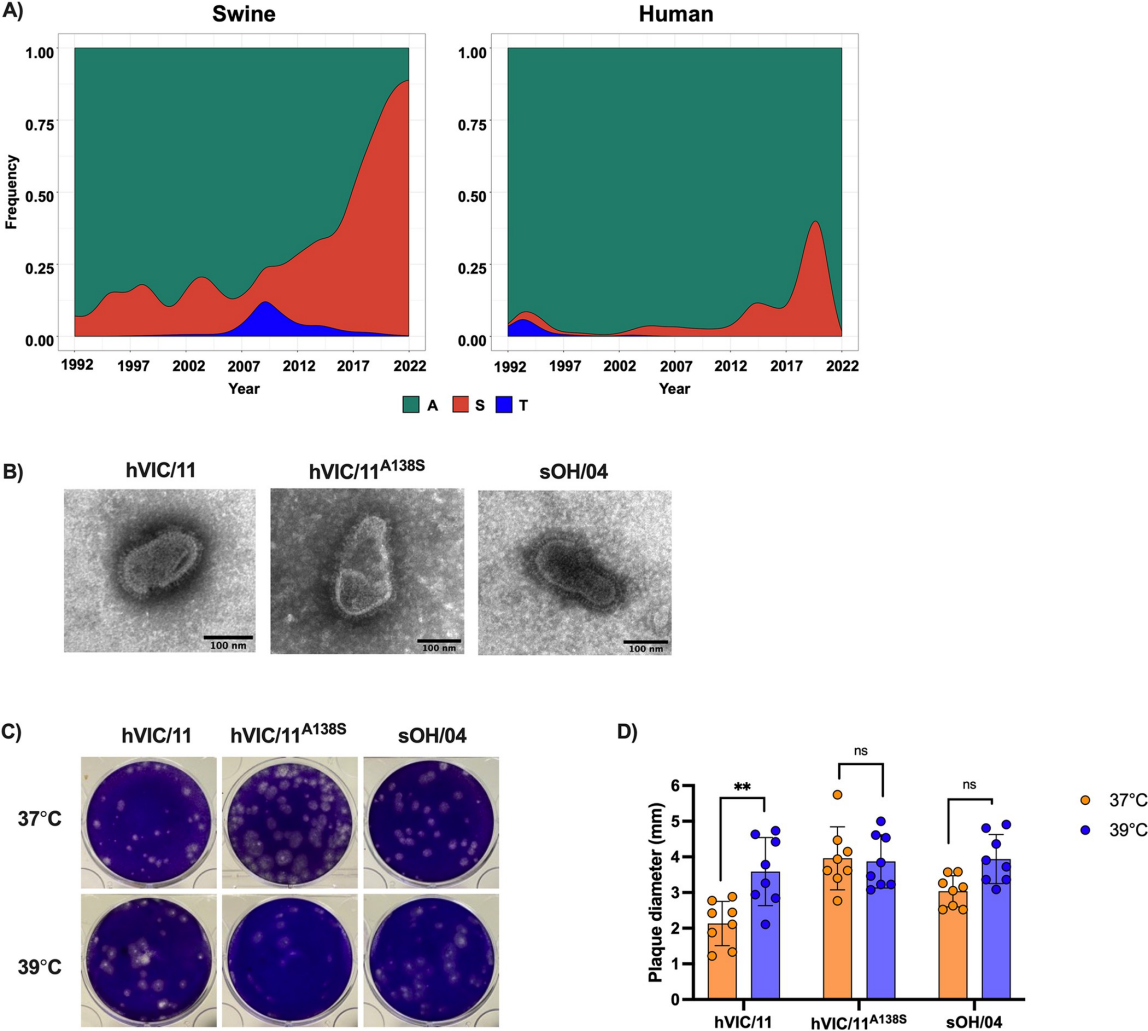
In vitro characterization of the hVIC/11^A138S^ virus. (A) Prevalence of the S138 residue in swine and human H3N2 FLUAV isolates reported from 1992 to 2022. Sequences were obtained from GISAID and aligned using ClustalW. (B) Representative electron microscopy pictures of sOH/04, hVIC/11, and hVIC/11^A138S^. Scale bar = 100 nm (C) Plaque morphology produced by sOH/04, hVIC/11, and hVIC/11^A138S^ in MDCK cells at 37 and 39°C. (D) Plaque sizes produced by the viruses at 37 and 39°C. Two independent experiments were performed in triplicates. Values represent the mean ± standard error of the mean (SEM). Statistical analysis was performed by two-way ANOVA. **p<0.005.

Previous *in vitro* experiments using differentiated primary swine tracheal cells showed a fitness advantage associated with the A138S change of the HA [35,36]. We analyzed the amino acids present at HA’s position 138 in 5,706 unique swine H3N2 sequences available on GISAID (Fig 1A). Before the spillover of the 2010.1 human-origin lineage into pigs, 80.3% of swine H3N2 isolates showed alanine (Ala; A138) in position 138 while serine (Ser; S138) was present in less than 15% of swine HA sequences (Fig 1A, left panel). However, after 2010, S138 showed a dramatic increase in detection frequency being present in 54.1% of swine isolates and, as of 2022, 96% of deposited sequences showed S138. When the frequency data were split between 1990.4 (another highly prevalent H3N2 FLUAV lineage of swine) and 2010.1 lineages (S1 Fig), we found that S138 was present in the 1990.4 lineage with a low frequency (10–20%) until 2019, when S138 became predominant, reaching 90% of sequences around 2020. Similarly, early 2010.1 sequences showed mainly A138 and S138 gradually increased reaching 100% frequency in 2022 and 2023. Conversely, human H3N2 viruses showed a strong preference for A138 (Fig 1A, right panel), and as of 2022, 98% of deposited sequences on GISAID showed A138, thus suggesting a potential advantage of the A138S mutation in the HA of swine H3N2 viruses for replication in pigs.

To assess the impact of this amino acid change on virus structure, virus morphology was evaluated (Fig 1B) in the context of a human-derived HA (hVIC/11 and hVIC/11^A138S^). For comparison purposes, a swine-adapted strain (sOH/04) was included in our analysis. Viruses were generated using reverse genetics carrying an isogenic backbone containing genes from the TRIG (PB2, PB1, PA, NP, NS) and H1N1pdm09 (M) lineages and the HA and NA genes from the human strain A/Victoria/361/2011 (hVIC/11), an hVIC/11 HA segment carrying the A138S mutation (hVIC/11^A138S^) and from the swine-adapted virus A/turkey/Ohio/313053/2004 (sOH/04). All viruses showed a spherical-like shape with an average diameter of 120 nm (Fig 1B), demonstrating that the A138S did not visibly alter the particle morphology. Further, viral plaque analysis showed no differences at either 37 or 39°C for sOH/04 and hVIC/11^A138S^ (Fig 1C and 1D), contrasting hVIC/11, which showed increased plaque sizes at 39°C. No significant differences were observed in virus replication in Madin Darby Canine Kidney (MDCK) cells among the 3 viruses evaluated at either 37 or 39°C (S2 Fig).

**Fig 2:**
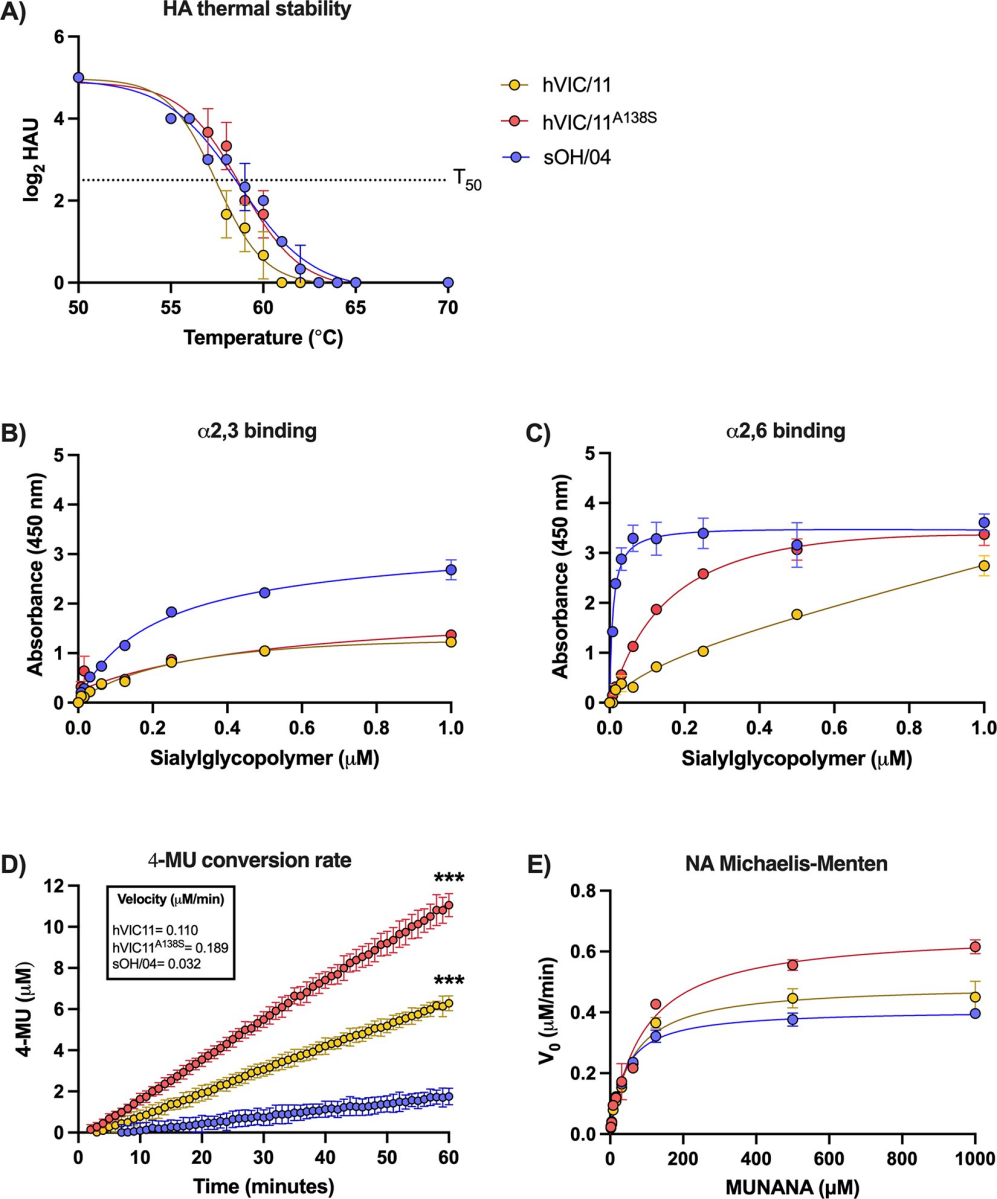
A138S increases HA thermal stability, binding for α2,6 receptors, and NA activity. (A) Thermal stability of sOH/04 (blue), hVIC/11 (yellow), and hVIC/11^A138S^ (red) was determined by incubating them at different temperatures for 1 hour. Data were fitted to a dose-response-inhibition non-linear fit. Receptor-binding affinity of sOH/04, hVIC/11, and hVIC/11^A138S^ for 3’SLN (B) or 6’SLN (C) was assessed by incubating the viruses with different concentrations of 3’SLN or 6’SLN. (D) NA activity was determined by normalizing the viruses at 10^4^ PFU/well in the presence of 100 μM MUNANA. Fluorescence was measured every 60 seconds for 1 hour and data was fitted to a linear regression model. (E) NA activity of the viruses was determined by normalizing based on NA activity. Viruses were incubated at different MUNANA concentrations for 1 hour and kinetic parameters (K_M_ and V_max_) were determined by fitting the data to the Michaelis-Menten equation. For all assays, two experiments were performed in triplicates. Values represent the mean ± SEM. Statistical analysis was performed by two-way ANOVA. ***p<0.0005.

To better understand the impact of the A138S mutation on the HA protein, we analyzed the thermal stability of the viruses (Fig 2A). The hVIC/11 showed reduced HA titers at lower temperatures when compared to sOH/04 and hVIC/11^A138S^, with a half-inactivation temperature (T_50_) of 57.4±0.2°C. The swine-adapted sOH/04 and hVIC/11^A138S^ virus had a T_50_ of 58.6±0.1°C and 58.7±0.3°C, respectively. We also analyzed the binding properties of the viruses using a high molecular weight sialylglycopolymer-based assay validated using mammalian- and avian-adapted viruses (S3 Fig) [37,38]. There were no differences between hVIC/11 and hVIC/11^A138S^ for α2,3 receptor binding (3’SLN, Fig 2B), while the swine-adapted sOH/04 virus had a higher affinity for 3’SLN. Noteworthy, hVIC/11^A138S^ exhibited an increased binding affinity to α2,6 receptors (6’SLN, Fig 2C) compared to hVIC/11, reaching similar levels as sOH/04.

Evaluation of NA sialidase activity using a MUNANA-based assay, and by normalizing each virus to 10^4^ plaque-forming units (PFU, Fig 2D) under MUNANA-saturated conditions (100 μM), showed that hVIC/11^A138S^ had the highest NA activity with a conversion rate of 0.189 μM/min, which was significantly higher than hVIC/11 (0.110 μM/min) and sOH/04 (0.032 μM/min). Results suggested that the A138S mutation in the HA protein influences NA enzymatic activity. We validated the assay’s specificity by oseltamivir inhibition (S4 Fig). These results were further confirmed by determining the NA kinetic parameters normalizing based only on NA activity. When the viruses were incubated with variable concentrations of MUNANA (Fig 2E), sOH/04 had the lowest *V*_*max*_ and *K*_*M*_ (0.408 μM/min and 39.25 μM respectively) whereas hVIC/11^A138S^ had the highest activity (*V*_*max*_ *=* 0.665 μM/min, *K*_*M*_ = 88.78 μM). The hVIC/11 showed an intermediate phenotype (*V*_*max*_ *=* 0.491 μM/min, *K*_*M*_ = 58.4 μM). Taken together, these results demonstrate that the A138S modulates the thermostability of the HA protein, increases affinity for α2,6-type receptors and affects NA activity.

### HA A138S improves transmission in pigs and infection of the lower respiratory tract

To evaluate the effect of the A138S mutation on transmission *in vivo*, 3-week-old pigs were inoculated with 3x10^6^ TCID_50_/pig of hVIC/11, hVIC/11^A183S^ or the swine-adapted sOH/04 (seeders, 3 pigs/virus, S5 Fig). Two days post-infection (dpi), 3 naïve pigs were introduced as contacts in each cage (contact 1), and the infection progressed for 3 more days. At 5 dpi seeders were humanely euthanized, and 3 new naïve pigs were introduced (contact 2). This cycle was repeated for a total of 4 contacts with contacts introduced at 3dpc each time. Tissues and BALF were collected from seeder pigs at 5dpi. In addition, nasal swabs were collected at 0 and 2dpi/3dpc from seeders and contact pigs.

FLUAV infection was evaluated by RT-qPCR from nasal swabs at 2dpi/3dpc (Table 1). sOH/04 was detected in all inoculated and contact animals throughout the study. Similarly, the hVIC/11^A138S^ virus was also detected among all contacts, contrasting with the hVIC/11 virus that was only detected in the seeders and contact 1 pigs, confirming the role of the A138S mutation in improving the transmissibility of hVIC/11.

**Table 1:** Virus detection in contact pigs infected with sOH/04, hVIC/11, and hVIC/11^A138S^. Number of FLUAV-positive contact pigs determined by RT-qPCR from nasal swab samples at 2dpi/3dpc (C1 = contact 1, C2 = contact 2, C3 = contact 3, C4 = contact 4).

Virus			
	sOH/04	hVIC/11	hVIC/11^A138S^
**Seeders**	3/3	3/3	3/3
**C1**	3/3	2/3	3/3
**C2**	3/3	0/3	3/3
**C3**	3/3	0/3	3/3
**C4**	3/3	0/3	3/3

**Fig 3:**
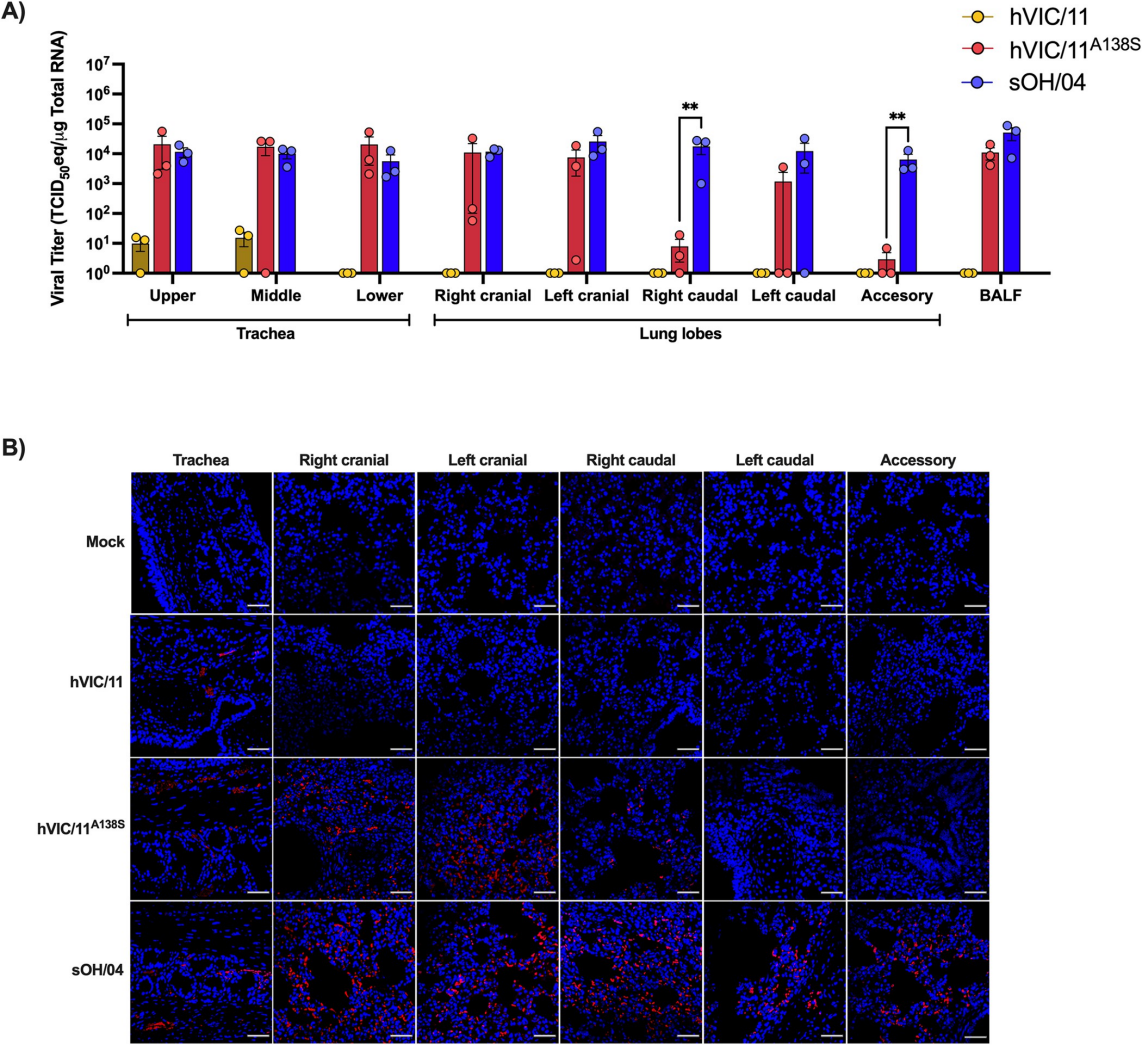
A138S improves infection of the lower respiratory tract of pigs. (A) Viral titers in different anatomical sections of the lungs of seeder pigs at 5 dpi (n = 3) normalized to 1μg total RNA. All statistical analyses were performed by two-way ANOVA. Values represent the mean ± SEM. *p<0.05, **p<0.005. (B) Influenza immunofluorescence staining in the respiratory tract of seeder pigs. Hemagglutinin (red) was detected using a polyclonal multi-H3 antibody and cell nuclei (blue) were stained with DAPI. The scale bar represents 50 μm.

Assessment of viral loads throughout the respiratory tract was performed by collecting different anatomical sections of the upper, middle, and lower trachea, right cranial lobe, left cranial lobe, right caudal lobe, left caudal lobe, and the accessory lobe from seeder pigs at 5 dpi (Fig 3A). vRNA was detected in all the collected tissues of all pigs in the sOH/04-infected group. Meanwhile, vRNA was detected in most tissues from the hVIC/11^A138S^-infected pigs with mean titers of 10^4^ TCID_50_eq/μg total RNA, except in the right caudal and the accessory lobe in which titers dropped to 10^1^ TCID_50_eq/μg total RNA. Titers in the left caudal lobe showed that 1 out of 3 pigs had virus in this lobe. Additionally, 2 out of 3 pigs showed lower titers in the right cranial lobe compared to sOH/04. Distinctively, vRNA loads in hVIC/11-infected pigs were only observed in the upper and middle trachea in 2 out of 3 seeder pigs with a mean titer of 10^1^ TCID_50_eq/μg total RNA. Viral infection was also confirmed by immunofluorescence (Fig 3B). Similar to viral RNA titrations, immunofluorescent imaging showed that the A138S mutation resulted in virus infection of the lower respiratory tract of pigs at 5 dpi. Conclusively, the results suggest the hVIC/11 containing the A138S mutation displayed an intermediate phenotype between the swine-adapted sOH/04 virus and the human hVIC/11 virus.

**Fig 4:**
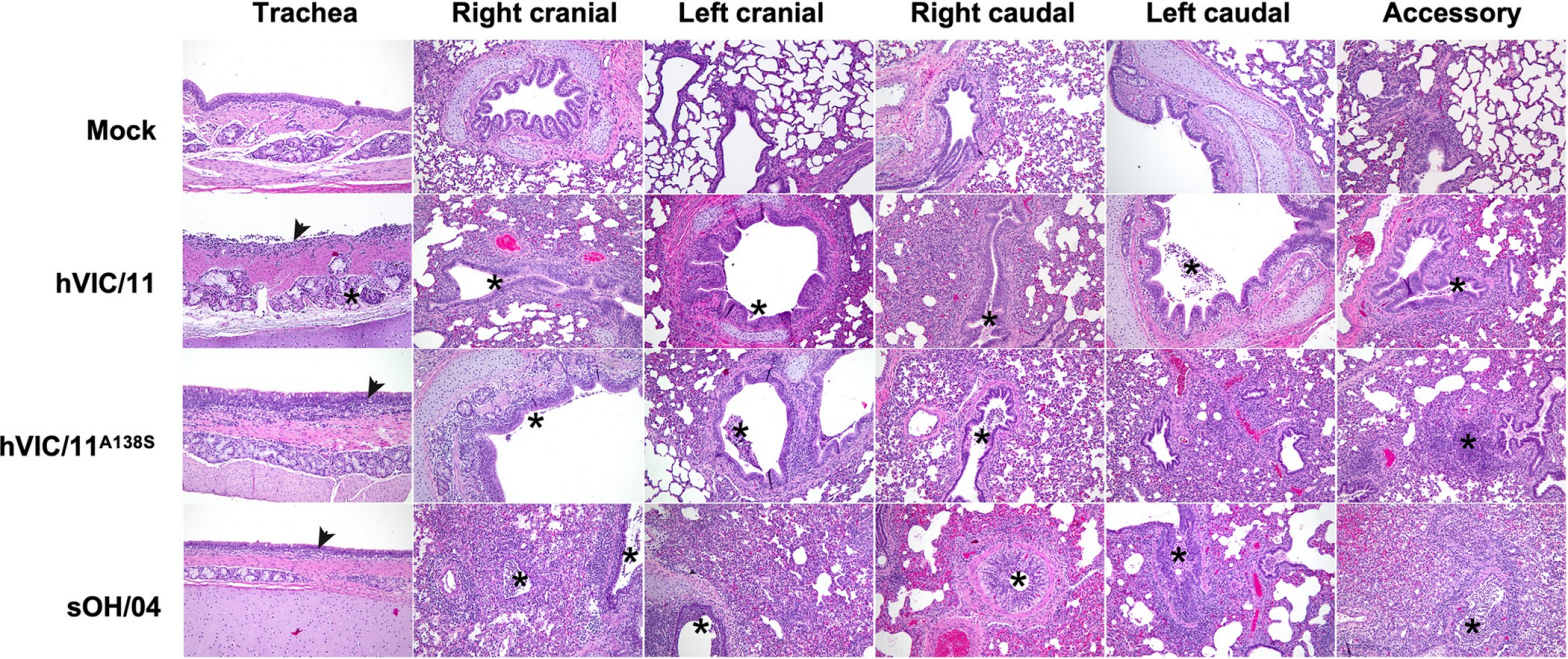
A138S does not affect histopathological findings in tracheas and lung lobes of infected pigs. Representative photomicrographs of trachea and lung sections of seeder pigs at 5 dpi, H&E, 10X. Mock group showing normal tissue sections. hVIC/11 group, trachea: epithelium was diffusely sloughing into the lumen (arrow) with a mild-moderate suppurative inflammation in the submucosal glands (asterisk). Lung: mild-moderate degree of suppurative bronchitis and bronchiolitis were present (asterisks). hVIC/11^A138S^ group, trachea: mild-moderate lymphohistiocytic inflammation expanded the lamina propria and effaced the mucosal epithelium. Lung: mild suppurative and catarrhal bronchitis and bronchiolitis (asterisks) were present with mild lymphohistiocytic cuffing of the airway. sOH/04 group, trachea: mild lymphohistiocytic tracheitis (arrow) was present. Lung: marked suppurative inflammation in the airways (asterisks)and adjacent alveoli.

Histopathological analysis revealed that the sOH/04 group exhibited moderate-severe necrotizing bronchiolitis with concurrent suppurative bronchitis, bronchiolitis, culminating into bronchiolitis obliterans (Fig 4). Moderate to severe microscopic lesions were present in the caudal lung lobe sections although, in these, they were principally centered on the airways. Only mild-moderate lymphohistiocytic tracheitis with mild multifocal epithelial degeneration and necrosis was observed in this sOH/04 group. The hVIC/11 group tracheas also presented mild-moderate lesions with only one section having moderate epithelial necrosis and suppurative inflammation of the submucosal glands. Lungs in this group had mild or mild-moderate suppurative bronchitis and bronchiolitis, contrasting the severe bronchiolitis observed in the sOH/04 group. The hVIC/11^A138S^ group had similar pulmonary and tracheal lesions to the hVIC/11 group. Concurrent catarrhal to suppurative bronchitis and bronchiolitis were accompanied by mild local epithelial degeneration, deciliation and sloughing into the lumen. Evidence suggests that the A138S mutation does not increase the tissue damage compared to the hVIC/11 virus.

To further understand the differences in FLUAV tropism, sialic acid receptor distribution in the respiratory tract of pigs was evaluated by staining α2,3 and α2,6 receptors using lectins (S6 Fig). Among multiple pigs, α2,6 receptors were predominant in the trachea, while α2,3 abundance increased in lower respiratory tract. α2,3 and α2,6 receptors were similarly distributed in all the pulmonary lobes which is in agreement with previous reports [39]. Taken together, evidence suggest that differences observed here may not be because of differences in receptor distribution.

### Viruses induced a distinct pattern of innate immune responses *in vivo*

**Fig 5:**
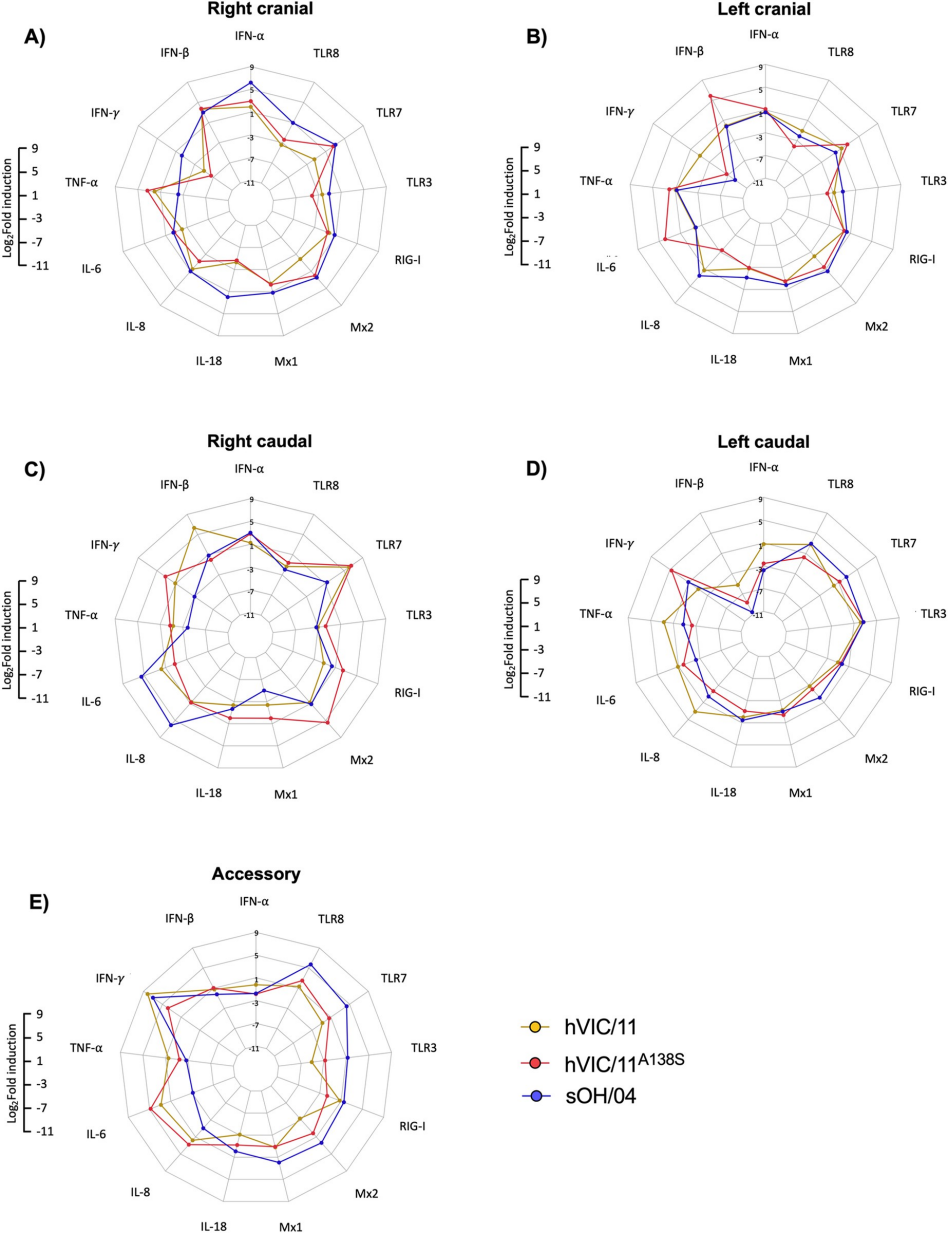
Distinct patterns of immune response are triggered by the viruses in different lung anatomical sections of pigs. Relative mRNA levels of pro-inflammatory cytokines, interferon-stimulated genes, and patter-recognition receptors. RNA was normalized to 1μg and gene expression was assessed by qPCR and normalized to RLP-19 expression in the (A) right cranial lobe, (B) left cranial lobe, (C) right caudal lobe, (D) left caudal lobe, and (E) accessory lobe. Values are shown as log_2_ fold induction of the mean between the seeder pigs (n = 3) of each group at 5dpi. Fold induction of each group was normalized to the non-infected negative control group.

To assess the expression of specific pro-inflammatory cytokines, interferon-induced genes, and pattern recognition receptors, RNA from each anatomical section of the lungs was extracted, gene expression was assessed by RT-qPCR, and fold induction was calculated by normalizing expression to the negative control group. In the right cranial lobe (Figs 5A and S7), sOH/04 induced the expression of numerous analyzed genes, being statistically significant TLR-7, Mx2, and IL-18. In contrast, hVIC/11^A138S^ showed a similar expression pattern as hVIC/11, which was characterized by a strong TNF-α and TLR-7 expression. In the left cranial lobe (Figs 5B and S8), there was a significant amount of IFN-γ repression within the sOH/04 and hVIC/11^A138S^-infected pigs, contrasting with hVIC/11-infected pigs where IFN-γ was not repressed. However, hVIC/11^A138S^-infected pigs displayed increased expression of IL-6 and IFN-β. For the right caudal lobe (Figs 5C and S9), sOH/04 significantly increased the expression of both IL-6 and IL-8, while hVIC/11 and hVIC/11^A138S^ induced a high level of TLR7 transcription. Individually, hVIC/11^A138S^ led to high expression of IFN-γ and Mx2, while hVIC/11 induced a strong IFN-β expression when compared to hVIC/11^A138S^, although the virus was not detected in this lobe at 5dpi (Fig 3). Expression patterns observed in the left caudal lobe differed (Figs 5D and S10), with all viruses strongly repressing IFN-β but most other genes did not exhibit major changes. Exceptions were seen for IFN-γ, which was slightly overexpressed by hVIC/11^A138S^ compared to hVIC/11; and IL-8 and TNF-α, which were slightly overexpressed by hVIC/11, however, these differences were not statistically significant. Lastly, sOH/04 induced the expression of almost all the PRRs analyzed in the accessory lobe (Figs 5E and S11). Meanwhile, hVIC/11 and hVIC/11^A138S^ overexpressed IL-6. In summary, our results showed that each virus induced a unique expression pattern in each lung lobe.

### Viruses induce a differential recruitment of immune cells to the lungs upon infection

**Fig 6:**
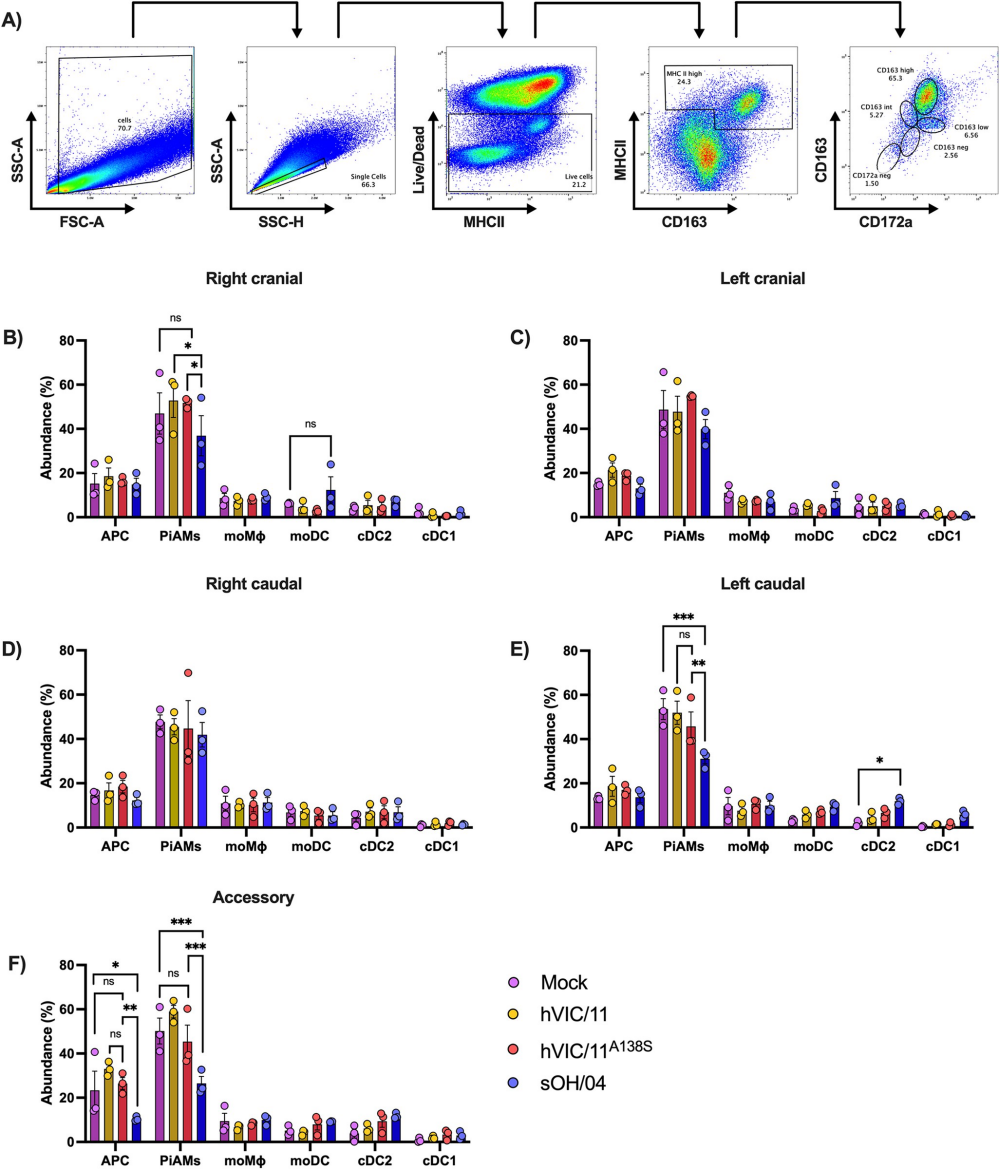
Distinct antigen-presenting cells, macrophages, and dendritic cells abundance induced after infection with influenza viruses in different lung anatomical sections of pigs. (A) Single-cell suspensions were labeled with anti MHCII, CD163, and CD172a antibodies and then analyzed by multi-color flow cytometry. Live cells from singlets were filtered and used to assess MHC II, CD163, and CD172a content. From the MHCII^high^ CD163^pos^ (antigen-presenting cells) population in lung tissue samples, cDC1 cells appear as CD172a^neg^CD163^neg^, cDC2 are CD172a^pos^CD163^neg^, moDC are CD172a^pos^CD163^low^, moMϕ are CD172a^pos^CD163^int^, and PiAMs are CD172aposCD163^high^. Abundance of APC, cDC1, cDC2, moDC, moMϕ, and PiAMs in the (A) right cranial lobe, (B) left cranial lobe, (C) right caudal lobe, (D) left caudal lobe, and (E) accessory lobe was quantified among total live cells (APC) or MHC^high^CD163^pos^ cells (cDC1, cDC2, moDC, moMϕ, and PiAMs). Values represent the mean ± SEM for seeder pigs (n = 3) in each group. Statistical analysis was performed by two-way ANOVA. *p<0.05, **p<0.005, ***p<0.0005.

To further characterize the host-immune response post-FLUAV infection of seeder pigs, cell populations that infiltrated in the different lung sections at 5 dpi were examined using flow cytometry and staining simultaneously for SLA class II DR (MHC II), CD163, and CD172a (Fig 6). This previously reported strategy enabled the detection of 5 different immune cell populations based on their CD163 content and CD172a presence gated on MHCII^high^CD163^pos^ cells (antigen-presenting cells, APC) (Fig 6A) [14]. These populations are CD172a^neg^CD163^neg^ (type 1 conventional dendritic cells, cDC1), CD172a^pos^CD163^neg^ (type 2 conventional dendritic cells, cDC2), CD172a^pos^CD163^low^ (monocyte-derived dendritic cells, moDC), CD172a^pos^CD163^int^ (monocyte-derived macrophages, moMϕ), and CD172a^pos^CD163^high^ (PAMs in BALF or interstitial PAMs in lung tissues, PiAMs). No changes were detected in the abundance of APC (MHCII^high^CD163^pos^) in the right cranial lobe (Figs 6B and S12A). However, the amount of PiAM (CD172a^pos^CD163^high^) was lower in sOH/04-infected pigs (36.9%) than the mock controls (46.9%). No significant differences between the infected groups and the mock control were observed in the remaining cell populations. Further, no differences were observed between groups in the left cranial lobe (Figs 6C and S12B) and the right caudal lobe (Figs 6D and S12C). A significant decrease of the PiAMs population was observed in the left caudal lobe in sOH/04-infected pigs (31.1%) compared to the mock group (53.6%) (Figs 6E and S12D). Similarly, we detected an increase in the cDC2 (CD172a^pos^CD163^neg^) population in the sOH/04-infected pigs (11.8%) compared to the mock (1.87%) in this lobe. No differences were observed between the mock and the other infected groups (hVIC/11 and hVIC/11^A138S^) in this lobe. In the accessory lobe (Figs 6F and S12E), sOH/04-infected pigs displayed a significant decrease in the number of APC cells (10.3%) compared to the mock (23.3%). No differences were observed between the mock- (50.16%), hVIC/11- (58.93%), and hVIC/11^A138S^-infected pigs (45.36%) for the PiAMs population in this lobe, while PiAMs were significantly reduced in sOH/04 -infected pigs (26.9%) compared to the mock control. Taken together, these results suggest that FLUAV infection with a well-adapted virus such as sOH/04 distinctively disrupts the PiAMs population in different sections of the lungs.

### The A138S mutation increases affinity for alveolar macrophages and enhances apoptosis induction

**Fig 7:**
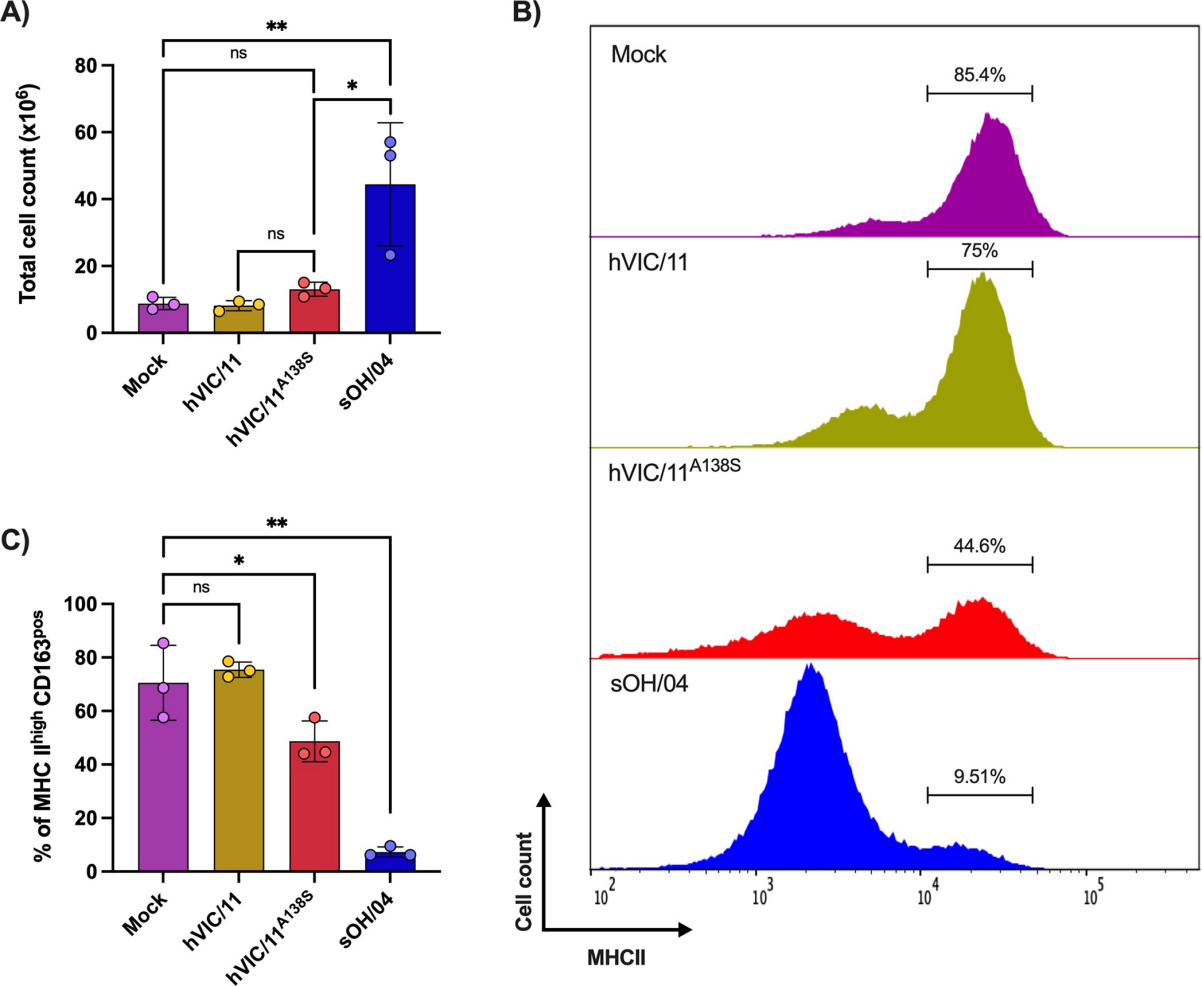
hVIC/11^A138S^ infection reduces the abundance of PAMs in BALF. **(**A) Total cell count in BALF samples from mock, hVIC/11, hVIC/11^A138S^, and sOH/04-infected pigs. (B) Representative histograms showing PAMs abundance (cell count, MHCII^high^CD163^pos^) in BALF samples. BALF samples were analyzed by multi-color flow cytometry and the MHCII content of the populations was quantified among total live cells. (C) Variation of PAMs abundance in mock, sOH/04, hVIC/11, and hVIC/11^A138S^-infected pigs quantified by flow cytometry. Values represent the mean ± SEM for seeder pigs (n = 3) per group. Statistical analysis was performed by two-way ANOVA. *p<0.05, **p<0.005.

Since sOH/04-infected pigs showed a decreased number of PiAMs, we next evaluated the effects of FLUAV infection on PAMs in BALF. First, differences in the total number of cells in BALF samples (Fig 7A) were assessed. Pigs infected with sOH/04 (4.4x10^7^ cells) displayed elevated cell counts in comparison to the mock (8.7x10^6^ cells), hVIC/11 (9.43x10^6^ cells), and hVIC/11^A138S^ (1.17x10^7^ cells). No differences were observed in the total cell count between the mock, hVIC/11, and hVIC/11^A138S^ groups. Following the strategy described above (Fig 6A), we further characterized the cell populations present in BALF by multi-color flow cytometry. Results showed that PAMs accounted for more than 75% of cells in BALF samples in both mock- and hVIC/11-infected pigs (Figs 7B, 7C, and S12F). Yet, the PAM population accounted for less than 50% in the hVIC/11^A138S^-infected pigs (44.6%). Similarly, we observed an increase of the MHCII^low^ CD163^neg^CD172a^neg^ population that increased proportionally with the reduction in PAMs percentage in BALF samples (Figs 7B and S12F). A more severe reduction was observed for the sOH/04-infected pigs, where the PAMs population was reduced to 9.51% (Figs 7B, 7C, and S12F).

**Fig 8:**
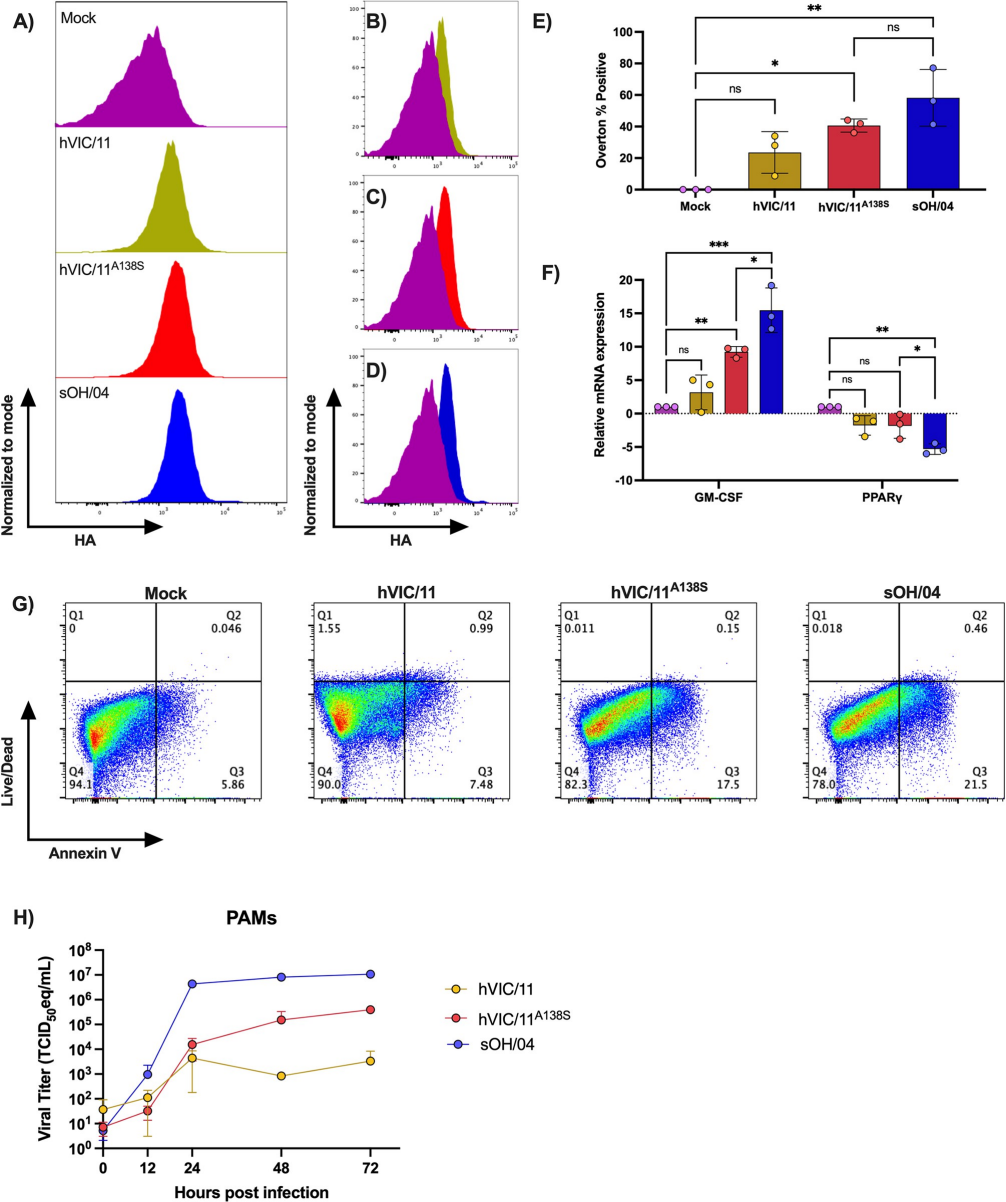
FLUAV infection of PAMs affects the expression of GM-CSF but not PPARγ expression and promotes apoptosis of alveolar macrophages. (A) PAMs collected from BALF samples of seeders at 5 dpi were stained for HA detection and analyzed by multi-color flow cytometry to detect the amount of MHCII^high^CD163^pos^ (PAMs) FLUAV -positive cells. Average histograms normalized to mode showing FLUAV -positive PAMs compared to the mock group for (B) hVIC/11, (C) hVIC/11^A138S^, and (D) sOH/04-infected seeder pigs (n = 3) per group. From the comparison of FLUAV-positive cells to the mock group, (E) the percentage of FLUAV-positive cells was determined using the Overton % method. (F) Relative mRNA levels of GM-CSF and PPARγ assessed by qPCR and normalized to RLP-19 expression in BALF samples. Values are shown as log_2_ fold induction of the mean between the seeder pigs (n = 3) of each group. (G) Flow cytometry analysis of apoptotic cells (Live/Dead^neg^Annexin V^pos^) at 12 hpi. (H) Growth kinetics of sOH/04 (blue), hVIC/11 (yellow), and hVIC/11^A138S^ (red) in 3D4/21 cells at 37°C. Two independent experiments were performed in triplicates each time. Values represent the mean ± SEM. Statistical analysis was performed by two-way ANOVA. *p<0.05, **p<0.005, ***p<0.0005.

Analysis of the amount of FLUAV- infected PAMs by flow cytometry by looking at the HA content on the cell surface among the MHCII^high^ CD163^pos^ population (Fig 8A) revealed that hVIC/11-infected pigs had the smallest % of HA-positive PAMs (23.56%) (Fig 8B and 8E). An increase of HA-positive cells was observed in hVIC/11^A138S^- and sOH/04-infected pigs (Fig 8C and 8D, respectively). sOH/04-infected pigs exhibited 60% of PAMs positive for HA, which was statistically higher compared to both hVIC/11 and the mock control (Fig 8E). Meanwhile, hVIC/11^A138S^ -infected pigs contained 40.63% of PAMs positive for HA which was statistically higher compared the mock control. Taken together, the A138S mutation showed an intermediate phenotype between the swine-adapted sOH/04 virus and the human hVIC/11 virus.

Next, the ability of the viruses to suppress the expression of key genes driving PAMs proliferation and immune activity was evaluated. Therefore, we looked at GM-CSF and PPARγ expression. GM-CSF is the main factor driving monocyte differentiation into PAMs *in vitro* and has been associated with PAM immune activity [30,40]. PPARγ is a transcription factor stimulated by GM-CSF; therefore, their expression will help to detect if FLUAV interferes with PAMs activity upstream or downstream the GM-CSF signaling. Analysis of GM-CSF expression in BALF samples showed increased expression upon FLUAV infection with all three viruses (Fig 8F); however, expression in hVIC/11-infected BALF showed no statistical differences compared to the mock. The expression of this gene was highly stimulated by sOH/04 infection, while hVIC/11^A138S^ displayed an intermediate phenotype. GM-CSF results contrast with PPARγ expression (Fig 8F), in which no major differences were detected between the mock, hVIC/11-, and hVIC/11^A138S^-infected pigs; however, PPARγ expression was strongly suppressed, with an expression 5 times lower than the mock control, in BALF samples from sOH/04-infected pigs. Overall, these results suggest that the A138S mutation increases FLUAV affinity for PAMs, but it is not enough to repress PPARγ expression as observed in sOH/04.

Finally, to confirm the ability of FLUAV to infect and induce death in PAMs, we used a commercial porcine alveolar macrophages cell line (3D4/21). When we looked at the ability of the viruses to induce apoptosis at 12 hpi (Fig 8G), we found that hVIC/11 (~7.5%) induced little apoptosis (Live/Dead^neg^Annexin V^pos^, Q3) in the cells compared to the mock control (~6%). However, an increased number of Annexin V^pos^ cells were found in the hVIC/11^A138S^ (~18%) and sOH/04 (~22%) infections. When we evaluated the growth kinetics of each virus in 3D4/21 cells (Fig 8H), sOH/04 showed the fastest replication, reaching a maximum titer of 10^7^ TCID_50_eq/mL at 24 hpi, which was significantly higher than hVIC/11^A138S^ that had a maximum titer of 10^5^ TCID_50_eq/mL at 72 hpi. In contrast, hVIC/11 showed an increase in titer at 24 hpi, but then it stopped replicating. Together, these results suggest that the increased apoptosis may be influenced by the increased replication ability of the swine sOH/04 compared to the human hVIC/11.

## Discussion

Understanding the mechanisms driving FLUAV evolution and adaptation to different species is critical for human and animal health. Human-origin FLUAV gene segments have been introduced in swine FLUAV strains, further expanding the genetic diversity of swine FLUAV [41,42]. Since the introduction of the TRIG constellation in the late 1990s, multiple human-to-swine spillover events have occurred, with increased frequency after the emergence of the pandemic H1N1 in 2009 [43]. Although this has contributed to the reassortment and maintenance of the human-origin internal genes [43,44], wholly human-origin viruses do not commonly persist in the swine population [44,45]. Interestingly, multiple HA and NA genes derived from human-seasonal H3N2 viruses have been introduced to the swine population leading to the emergence of distinct phylogenetic clades [46–49]. In this work, we have found that the adaptation of human-origin FLUAV HA to pigs increases affinity for the lower respiratory tract and leads to PAM depletion, possibly by triggering apoptosis, which might be a critical step for adaptation of human viruses to the swine host.

The H3 HA A138S mutation is prevalent in swine HA genes, and this increased significantly after the emergence of the H3 2010.1 lineage. This mutation has been associated with the adaptation of avian-origin H3, H6, and H7 to mammalian receptors [50] and increased infectivity of H3N2 viruses in swine respiratory epithelial cells [36]. However, no previous studies have evaluated the in-depth impact of this amino acid change on the virus adaptation to the swine respiratory tract and subsequent transmission between animals. Previously, we and others showed that the A138S mutation exhibits increased replication and binding to swine tracheal cells [35,36]. Using a reassortant hVIC/11^A138S^ virus, we detected a small increase in the HA thermostability but observed that this mutation does not affect viral morphology (Fig 1) or replication in MDCK cells. Increased HA thermostability suggests the virus retains biological activity at higher temperatures than the original hVIC/11 virus (Fig 2).

NA activity of the viruses revealed that the swine-adapted sOH/04 virus exhibited decreased activity, characterized by a reduced *V*_*max*_ and *K*_*M*_ compared to both hVIC/11 and hVIC/11^A138S^. A previous report showed that viruses with low NA activity are not inhibited by swine mucus, suggesting they are potentially transmissible among pigs but not humans [51]. This result contrasts with the activity of the hVIC/11^A138S^ virus that showed increased NA activity compared to sOH/04 and hVIC/11. Nonetheless, *K*_*M*_ for hVIC/11^A138S^ was almost doubled when compared to sOH/04, which means the virus needs two times more substrate to reach *V*_*max*_ than sOH/04. Hence, hVIC/11^A138S^ NA seems to have a decreased substrate affinity but enhanced catalytic activity compared to sOH/04 NA. This is especially interesting since the NA gene from both hVIC/11 and hVIC/11^A138S^ is the same, but differed significantly in NA activity, suggesting that mutations in the HA protein might modulate NA activity. Indeed, when we analyzed the HA affinity for α2,6 sialic acid receptors, hVIC/11^A138S^ displayed higher affinity than hVIC/11, with no differences compared to sOH/04 at high concentrations of 6’SLN (Fig 2). This could explain an increased NA activity without disrupting the functional balance between HA avidity and NA activity, considering that hVIC/11^A138S^ NA has less affinity for the receptor than sOH/04. However, more experiments beyond the scope of this study are required to confirm this hypothesis.

hVIC/11 showed reduced transmission *in vivo* and was not detected after contact 1 pigs, contrasting with hVIC/11^A138S^ and sOH/04 that efficiently transmitted through 4 subsequent transmission events. Therefore, the data supports our previous results that the A138S mutation increases viral replication and transmissibility in pigs. The A138S mutation exhibited efficient virus replication in most of the seeders’ upper and lower respiratory tracts, displaying a pattern similar to sOH/04 and differing from hVIC/11, which failed to infect the lower respiratory tract by 5 dpi. Numerous studies have demonstrated that efficient transmission of FLUAV is associated with enhanced replication in the lungs of ferrets and mice [52–55]. However, a recent study observed that upper respiratory tract infection is critical for onward transmission in the ferret model [56]. Nonetheless, the lack of transmission of hVIC/11, despite active replication in the nose and trachea, consistently with what has been shown previously [7], suggests a potential role of the lower respiratory tract infection of FLUAV in pigs. Since all the viruses possessed the same internal genes (TRIG backbone), the ability of the hVIC/11^A138S^ virus to infect the lower respiratory tract is most likely from a direct effect of this HA amino acid substitution, potentially linked to enhanced entry into swine cells induced by an increased affinity for α2,6 receptors. However, it cannot be discarded that hVIC/11 infected the lower respiratory tract and was cleared faster than sOH/04 and hVIC/11^A138S^, therefore impeding detection by the time of tissue collection.

Induction of the cellular immune response by FLUAV infection varied among different anatomical sections of the lungs but was not necessarily associated with virus detection in each lung section (Figs 3 and 5). Most of the analyzed cytokines were upregulated in the lungs of sOH/04-infected pigs, although TNF-α was down-regulated in almost all lobes except the left cranial lobe. Interestingly, TNF-α was not inhibited by hVIC/11^A138S^ in the right cranial, right caudal, and accessory lobe. TNF-α has been demonstrated to exert a potent antiviral activity in the lungs [57]; therefore, lower virus replication in these lung lobes could be due to overexpression of this cytokine. In addition, we detected higher expression of IFN-γ in the left caudal and right caudal lobe of hVIC/11^A138S^-infected pigs compared to sOH/04 coupled with elevated levels of IFN-β in the accessory lobe. These elevated levels of IFN coincided with limited virus replication in similar tissues. Considering that type I and II IFN are potent FLUAV antivirals [58], the results are consistent with the idea that FLUAV viruses need to inhibit expression of host-specific IFN to efficiently replicate in the lungs of a particular host. However, it must be noted that our study design only captures the expression profile at 5 dpi, and changes in the expression of certain genes and the distribution of the viruses in the respiratory tract before the time of collection may affect the outcome at 5 dpi. Further, since the number of animals used in this study was small and pigs were inoculated intratracheally and intranasally, we must exercise caution interpreting gene expression profiles of each individual lobe as the inoculation method could cause differences in virus deposition in the lungs that could affect gene expression and might differ from what is observed in a natural infection.

FLUAV infection of the lungs considerably impacted the cell populations in different lobes. We observed that sOH/04 infection decreased the presence of PiAMs in the right cranial, left caudal, and accessory lobe, but hVIC/11 and hVIC/11^A138S^ did not affect this population. Interestingly, hVIC/11^A138S^ showed reduced infection in some of these lobes, specifically exhibiting a trend for reduced titer in the left caudal lobe when compared to sOH/04 and no virus replication in the accessory lobe as demonstrated by RT-qPCR and immunofluorescence analysis at 5dpi (Fig 3). These results suggest that PiAMs may limit FLUAV replication in addition to IFN and TNF-α; therefore, the adaptation of human viruses may involve the ability to deplete PiAMs. Hence, the role of both PiAMs, which are phenotypically distinct from PAMs [14] in terms of gene expression profiles and morphology, during FLUAV infection deserves further investigation in future studies.

The cell content in BALF samples revealed that sOH/04 infection recruits a large number of cells to the lungs, noted from the ten-fold increase in the total number of cells compared to the mock group (Fig 7). The increased infiltration of cells is due to the recruitment of MHCII^low^CD163^neg^ cells, most likely neutrophils, and T cells, as has been previously reported [59–61]. The substantial increase in the percentage of neutrophils in the lungs could be due to the ability of the viruses to induce expression of IL-8, a cytokine known to function as a neutrophil chemoattractant [62,63]. We also detected that sOH/04 efficiently represses IFN-β expression in most of the lobes, which has been reported to act as a repressor of neutrophil infiltration [64, 65]. Notably, results for hVIC/11^A138S^ contrasted with sOH/04 as it failed to repress IFN-β and did not result in an elevated recruitment of cells. When taken together with IL-8 upregulation, the results suggest that hVIC/11^A138S^ infection leads to the recruitment of neutrophils to the site of infection but at lower levels than sOH/04, as shown by our flow cytometric analysis and represented as an increase of MHCII^low^ CD163^neg^ cells (Fig 7B).

The content of PAMs in BALF samples represented about 70% of the total cells in the mock- and hVIC/11-infected groups and was significantly reduced in sOH/04- and hVIC/11^A138S^-infected pigs (Fig 7), consistent with previous reports showing the depletion of AMs after FLUAV infection in mice [66]. The role of AMs during FLUAV infection is still debated; while some reports have shown active replication of FLUAV in these cells, others have demonstrated unproductive viral replication [34]. Nonetheless, previous reports showed little to no cell death after infection with human-adapted H3N2 viruses in human and porcine AMs [22,34,67], while infection of PAMs with swine-adapted viruses resulted in effective infection and cell death [26, 68]. Here, we showed that the number of influenza-positive PAMs increased with the level of adaptation to the swine host, with sOH/04 showing the highest number of infected cells. Our results suggest, in accordance with others [69], that adaptation to the swine host leads to the increased ability to infect, replicate, and induce apoptosis in PAMs (Fig 8). We have previously shown that this mutation increases binding and replication in differentiated swine tracheal cells [35], most likely due to enhanced affinity for α2,6 receptors. This could also explain the higher affinity of swine-adapted strains to PAMs. Here, we showed that infection with a virus that is highly adapted to pigs (sOH/04) resulted in downregulation of PPARγ. This receptor acts as a crucial transcription factor that promotes monocytes differentiation into AMs *in vivo* and *in vitro* [30]. Additionally, it has been reported to be an important inflammation modulator by limiting the expression of various pro-inflammatory cytokines [70]. In the context of FLUAV infection, PPARγ expression has been shown to reduce tissue damage and death of infected mice [71,72]. Further, previous reports have demonstrated that FLUAV inhibits PPARγ expression, which was associated with worsened lung injury in vivo [71], in accordance with our histopathological results for pigs infected with sOH/04 (Fig 4). Interestingly, GM-CSF expression was not repressed, and, considering that PPARγ expression is GM-CSF dependent, it is most likely that FLUAV interferes downstream of the GM-CSF signaling. The JAK/STAT pathway mediates GM-CSF signaling [40], which ultimately leads to STAT5 phosphorylation and translocation into the nucleus, where it mediates transcription of a variety of genes, including PPARγ [73]. It is possible that FLUAV disrupts the PAPRγ expression by inhibiting the JAK/STAT pathway due to the STAT-dependency in PAPRγ expression [74–77]. Here, the increased suppression of PAPRγ found in sOH/04-infected pigs is most likely due to the increased number of infected PAMs in these animals. This is supported by our data showing better infection and replication of PAMs by sOH/04 (Fig 8). Additionally, Annexin V staining revealed that both hVIC/11^A138S^ and sOH/04 induce apoptosis in infected PAMs. These findings, together with PAPRγ repression, could explain why sOH/04 is more efficient at depleting PAMs than hVIC/11^A138S^. While a relationship between viral fitness and PAMs infection was observed, future studies beyond the scope of the present report are needed to further understand the impact of PAMs depletion on the pigs’ innate and adaptative immune responses against FLUAV. Similarly, the effects of PPARγ suppression should be further studied to better understand its role in PAMs proliferation and anti-FLUAV activity, which could not be assessed with our experimental design.

Overall, our study indicates that the A138S mutation broadly impacts the virus phenotype, HA thermostability, NA activity, HA receptor affinity, host range, and tissue tropism. Notably, viruses carrying this mutation replicate more efficiently in the lower respiratory tract of pigs, possibly due to an increased α2,6 affinity and increased affinity for PAMs. Infection of pigs with swine-adapted viruses depleted PAMs at 5 dpi most likely by triggering apoptosis in infected cells but it might also disrupt their immune activity and proliferation by repressing the expression of PPARγ.

## Materials and methods

### Ethics statement

Animal studies were reviewed and approved by the Institutional Animal Care and Use Committee (IACUC) at the University of Georgia (protocol A2019 03-031-Y3-A9). Studies were conducted under biosafety level 2 containment and following the Guide for the Care and Use of Agricultural Animals in Research and Teaching.

### Cells and viruses

Madin-Darby canine kidney (MDCK) cells were maintained in Dulbecco’s Modified Eagles Medium (DMEM, Sigma-Aldrich, St Louis, MO) supplemented with 10% fetal bovine serum (FBS, Sigma-Aldrich, St Louis, MO), 2mM L-glutamine (Sigma-Aldrich, St Louis, MO), and 1% antibiotic/antimycotic (Sigma-Aldrich, St Louis, MO). 3D4/21 cells were maintained in Roswell Park Memorial Institute media (RPMI-1640, Sigma-Aldrich, St Louis, MO) supplemented with 10% FBS, 2mM L-glutamine, 1% antibiotic/antimycotic, 1 mM non-essential amino acids (Sigma-Aldrich, St Louis, MO), and 1 mM sodium pyruvate (ThermoFisher Scientific, Waltham, MA). All cell lines were cultured at 37°C under 5% CO_2_. Viruses used in this study were: a reassortant carrying seven genes from A/turkey/Ohio/313053/2004 and the pandemic H1N1pdm09 (A/California/04/09) matrix gene (sOH/04); a reassortant carrying the HA and NA genes from A/Victoria/361/2011 and internal genes from sOH/04 (hVIC/11); and hVIC/11^A138S^ which only differs from hVIC/11 by an A to S amino acid substitution at position 138 in the HA gene (H3 nomenclature). Notably, the PB2, PB1, PA, NP, M, and NS genes were the same for all viruses. These viruses have been previously reported by our group [35]. Viral titers were determined by TCID_50_ using the Reed and Muench method [78].

### Electron microscopy

Viruses were adsorbed for 5 minutes in formvar-carbon-coated copper grids (ThermoFisher Scientific, Waltham, MA). After adsorption, samples were fixed with 0.7% glutaraldehyde (Sigma-Aldrich, St Louis, MO) for 5 minutes at room temperature. After, samples were negatively stained with 3% phosphotungstic acid pH 7.0 (Sigma-Aldrich, St Louis, MO) for 60 seconds. Finally, the excess stain was drained, and the grids were dried on filter papers. Viruses were imaged using a JEOL JEM1011 transmission electron microscope (JEOL USA, Peabody, MA) at 80 kV.

### *In vitro* growth kinetics

MDCK cells were seeded in Opti-MEM (Life Technologies, Carlsbad, CA, USA) and incubated overnight or until a 70–80% confluency was reached. Cells were infected at a multiplicity of infection (MOI) of 0.01 for 1 hour at 37°C or 39°C. Immediately after, plates were washed three times with phosphate-buffered saline (PBS) and fresh Opti-MEM containing 1 μg/ml of tosylsulfonyl phenylalanyl chloromethyl ketone (TPCK)-treated trypsin (Worthington Biochemicals, Lakewood, NJ) was added. Timepoints were collected at 0, 12, 24, 48, and 72 hours post-infection (hpi). Viral RNA was extracted using the MagMax-96 AI/ND viral RNA isolation kit (ThermoFisher Scientific, Waltham, MA) according to the manufacturer’s instructions. A one-step real-time quantitative PCR (RT-qPCR) using the Quantabio qScript XLT One-Step RT-qPCR ToughMix kit (Quantabio, Beverly MA) targeting the M segment was used to determinate viral titers. The reaction master mix was prepared by mixing 1X Quantabio master mix, 0.5 μM of each primer, 0.3 μM TaqMan probe and 5 μL of RNA. Finally, viral titers were calculated according to a standard curve of an exact match of virus stock of known titer based on a TCID_50_ equivalent (TCID_50_eq/mL) standard curve.

Growth kinetics in 3D4/21 cells were performed by infecting cells at MOI of 0.01 for 1 hour at 37°C. After infection, cells were washed three times with PBS and supplemented with fresh infection media (RMPI-1640, 0.3% BSA, 2 mM L-glutamine, 1% antibiotic/antimycotic, 1 mM non-essential amino, 1 mM sodium pyruvate, and 250 ng/mL TPCK-treated trypsin).

### Plaque assay

MDCK cells were seeded in 6-well plates at 10^6^ cells/well and were incubated overnight at 37°C or until a 100% confluency before use. The next day cells were infected with 10-fold serial dilution of the viral stock for 1 hour at 37°C. After infection, cells were washed three times with PBS and overlayed with Opti-MEM containing 0.8% Avicel. Plates were incubated for 72 hours at 37°C or 39°C. Finally, cells were fixed for 1 hour with 37% formaldehyde (Sigma-Aldrich, St Louis, MO), rinsed twice with PBS, and stained for 15 minutes with 0.5% crystal violet in 20% methanol.

### Thermal stability

The thermal stability of the viruses was assessed by normalizing them to 32 HAU in PBS. Then, samples were incubated for 1 hour at the indicated temperatures. Afterward, samples were immediately placed in ice, and HA titers were measured using 0.5% turkey red blood cells.

### NA enzymatic activity

NA sialidase activity was measured as previously described by [79]. Briefly, viruses were diluted, and the dilution of choice was the one that met the following parameters: within the linear range and a saturated 2′-(4-Methylumbelliferyl)-α-D-N-acetylneuraminic acid sodium salt hydrate (MUNANA, Sigma-Aldrich, St Louis, MO) concentration. The chosen dilution was then used to calculate NA kinetic constants by performing kinetics for 60 minutes at 37°C at 1000, 500, 250, 125, 62.5, 31.25, 16.63, 7.81, 3.91, and 1.95 μM MUNANA. Fluorescence was measured every 60 seconds at excitation and emission wavelengths of 360 nm and 460 nm, respectively, using a Synergy HTX Multi-Mode Microplate Reader (Agilent BioTek, Santa Clara, CA).

The inner filter effect was corrected by measuring MUNANA absorbance at 4-Methylumbelliferone (4-MU) emission wavelength at different concentrations. Using the corrected fluorescence, 4-MU production over time was calculated using a standard curve and data was fitted to the Michaelis-Menten equation:

$$V_{0}=\frac{V_{max}*[S]}{K_{m}+[S]}$$

### Hemagglutinin solid-phase binding assay

A direct solid-phase binding assay using sialylglycopolymers was used as described by [80]. Briefly, viruses were pelleted by ultracentrifugation (28,000 rpm for 3 hours) through a 20% sucrose cushion and resuspended in TNE buffer (0.01M Tris, 0.001M EDTA, 0.1 M NaCl, pH 7.2). Purified viruses were normalized to 128 HAU in PBS and incubated overnight at 4°C in fetuin-coated 96-well plates (ThermoFisher Scientific, Waltham, MA). Plates were then washed three times with 0.02% Tween-80 (Sigma-Aldrich, St Louis, MO) in PBS (washing buffer) and blocked with 0.1% desialylated BSA (BSA-NA) in PBS (blocking solution) for 2 hours at room temperature. After blocking, plates were washed three times with washing buffer and 2-fold serial dilution of Neu5Acα2-3Galβ1-4GlcNAcβ-PAA-biotin (α2,3, 3’SNL, Glycotech, Gaithersburg, MD) or Neu5Acα2-6Galβ1-4GlcNAcβ-PAA-biotin (α2,6, 6’SLN, Lectinicity Holding, Moscow, Russia) in reaction buffer (0.02% Tween-80, 0.1% BSA-NA, and 2 μM oseltamivir in PBS) were added. Plates were incubated for 1 hour at 4°C. After, plates were washed five times with washing buffer and incubated with a 1:1,000 dilution of HRP-conjugated streptavidin (ThermoFisher Scientific, Waltham, MA) in reaction buffer for 1 hour at 4°C. Finally, plates were washed five times and then incubated with TMB (ThermoFisher Scientific, Waltham, MA) for 10 minutes at room temperature and the reaction was stopped using a stop solution (ThermoFisher Scientific, Waltham, MA). Absorbance was measured at 450 nm using a Synergy HTX Multi-Mode Microplate Reader.

### *In vivo* studies

3-weeks-old healthy cross-bred pigs were obtained from Midwest Research Swine Inc (Gibbon, MN) and housed in animal biosafety level 2 (BSL2) facilities at the University of Georgia. After a 7-day acclimatation period, pigs were bled to confirm the absence of anti-FLUAV antibodies by ELISA (IDEXX, Westbrook, ME), and randomly distributed into four groups of three pigs each and challenged intranasally and intratracheally with 3x10^6^ TCID_50_/pig of either sOH/04, hVIC/11 or hVIC/11^A138S^ under anesthesia using a cocktail of ketamine (6 mg/kg), xylazine (3 mg/kg), and telazol (6 mg/kg). Pigs were observed daily for clinical signs, and nasal swabs were collected at 0, 2, and 5 days post-infection (dpi). At 2 dpi, a new set of 3 pigs was introduced in the same housing as the inoculated pigs and nasal swabs were collected at 0, 3, and 6 dpc. At 5 dpi/6 dpc, pigs were anesthetized and humanely euthanized by an intravenous pentobarbital overdose (Euthasol, 200 mg/kg), and a new set of contacts was introduced. Upper, middle, and lower trachea, lung lobes (right cranial, left cranial, right caudal, left caudal, and accessory), and bronchoalveolar lavage fluid (BALF) samples were collected post-mortem and stored at -80°C for virus titration and gene expression analysis. All pigs were determined to be negative for Porcine circovirus type 2 (PCV2), Porcine reproductive and respiratory syndrome virus (PRRSV), and Mycoplasma hyopneumoniae by qPCR (Table 2) of BALF samples.

**Table 2:** Primer sequences used for gene expression analysis and pathogens detection from lung tissue and BALF samples.

Primer	Sequence (5’-3’)	Reference
RPL-19	F: AACTCCCGTCAGCAGATCC R: AGTACCCTTCCGCTTACCG	[82]
IL-1β	F: AGAAGAGCCCATCGTCCTTG R: GAGAGCCTTCAGCTCATGTG	
IL-6	F: ATCAGGAGACCTGCTTGATG R: TGGTGGCTTTGTCTGGATTC	
IL-8	F: TCCTGCTTTCTGCAGCTCTC R: GGGTGGAAAGGTGTGGAATG	
IFN-α	F: GGCTCTGGTGCATGAGATGC R: CAGCCAGGATGGAGTCCTCC	
IFN-β	F: ATGTCAGAAGCTCCTGGGACAGTT R: AGGTCATCCATCTGCCCATCAAGT	
TNF-α	F: CCAATGGCAGAGTGGGTATG R: TGAAGAGGACCTGGGAGTAG	
IFN-γ	F: GCTCTGGGAAACTGAATGAC R: TCTCTGGCCTTGGAACATAG	
iNOS	F: GAGAGGCAGAGGCTTGAGAC R: TGGAGGAGCTGATGGAGTAG	
Mx1	F: AGTGTCGGCTGTTTACCAAG R: TTCACAAACCCTGGCAACTC	
Mx2	F: CCGACTTCAGTTCAGGATGG R: ACAGGAGACGGTCCGTTTAC	
PKR	F: GACATCCAAAGCAGCTCTCC R: CGCTCTACCTTCTCGCAATC	
RIG-I	F: CGACATTGCTCAGTGCAATC R: TCAGCGTTAGCAGTCAGAAG	
TLR-3	F: CCTGCATTCCAGAAGTTGAG R: TGAGGTGGAGTATTGCAGAG	
TLR-7	F: TCAGCTACAACCAGCTGAAG R: CAGATGTCGCAACTGGAAAG	
TLR-8	F: AGCGCGGGAGGAGTATTGTG R: GCCAGGGCAGCCAACATAAC	
IL-18	F: TCCTTTTCATTAACCAGGGACATC R: GGTCTGAGGTGCATTATCTGAACA	[83]
GM-CSF	F: GCAGCATGTGGATGCCATCA R: GCTCCTGGGGGTCAAACATTTC	This work
PPARγ	F: TCCAGCATTTCCACTCCACACT R: GAATAAGGCGGGGACACAG	[84]
PCV2	F: ATAACCCAGCCCTTCTCCTACC R: GGCCTACGTGGTCTACATTTCC	[85]
PRRSV	F: AAACCAGTCCAGAGGCAAGG R: GCAAACTAA ACTCCACAGTGTAA	[86]
Mycoplasma	F: GTCAAAGTCAAAGTCAGCAAAC R: AGCTGTTCAAATGCTTGTCC	[87]

### Tissue preparation for FLUAV titration and nasal swab virus titration

Lung sections were homogenized using the Tissue Lizer II (Qiagen, Gaithersburg, MD) by adding 1 mL of PBS to each tube containing the sample and a Tungsten carbide 3 mm bead (Qiagen, Gaithersburg, MD) and then homogenizing for 10 minutes at 30 Hz. RNA from tissue and nasal swab samples was then extracted using the MagMax-96 AI/ND viral RNA isolation kit. Tissue samples were then normalized to 1 μg total RNA in 20 μL of nuclease-free water while RNA extracted from nasal swabs was used directly in the RT-qPCR reaction. The one-step RT-qPCR was performed using the Quantabio qScript XLT one-Step RT-qPCR ToughMix kit as described above and FLUAV TCID_50_ equivalent per μg of total RNA titers (TCID_50_eq/μg total RNA) in tissue sections was calculated according to a standard curve of an exact match of virus stock of known titer.

### Tissue immunofluorescence and histopathology

Lung sections were collected in 10% neutral-buffered formalin and paraffin-embedded. For immunofluorescence detection of FLUAV and sialic acid receptors, paraffin-embedded sections were deparaffinized as previously described [81] with minor modifications. Briefly, tissue slides were deparaffinized and rehydrated for subsequent heat-induced antigen retrieval in citrate buffer (10 mM sodium citrate, pH 6.0) for 40 minutes. Rehydrated samples were then permeabilized for 10 minutes with 0.3% TritonX-100 and blocked for 1 hour with 5% bovine serum albumin in PBS. FLUAV was detected using a primary anti-Multi-Hemagglutinin (H3N2) polyclonal antibody (eEnzyme, Gaithersburg, MD) followed by a secondary Alexa 647-conjugated anti-rabbit antibody in a 1:1,000 dilution in PBS containing 0.5 μg/mL 4’,6-diamine-2- phenylindole (DAPI, Sigma-Aldrich, St Louis, MO) for 1 hour each. Finally, tissues were washed 5 times with PBS and mounted on glass slides with mounting medium (Vector Laboratories, Newark, CA). Sialic acid receptors were detected by incubation of previously blocked samples with fluorescein-conjugated *Sambucus nigra* agglutinin (SNA) and biotin-conjugated *Maackia amurensis* agglutinin (MAL II) lectins (Vector Laboratories, Newark, CA) in a 1:250 dilution in PBS for 30 minutes followed by a 30-minute incubation with Alexa 594-conjugated streptavidin for MAL II detection (ThermoFisher Scientific, Waltham, MA). Samples were then permeabilized and incubated with 0.5 μg/mL DAPI for 15 minutes. Slides were imaged using a Nikon A1R confocal microscope (Nikon, Melville, NY).

A duplicate 3.5 μm section was processed for routine histopathology with hematoxylin and eosin staining (HE). Microscopic lesions were evaluated by a veterinary pathologist blind to treatment groups.

### Gene expression analysis

To assess the expression level of different cytokines, interferon-induced genes, and pattern recognition receptors, RNA from tissue and BALF samples was extracted using the MagMax-96 RNA isolation kit (ThermoFisher Scientific, Waltham, MA) and samples were normalized to 1 μg RNA/reaction. Contaminant genomic DNA was eliminated by treatment with the RQ1 RNase-free DNase (Promega, Madison, WI). DNA-free RNA was reverse transcribed using the M-MLV reverse transcriptase (Promega, Madison, WI) and oligo(dT) primers according to the manufacturer’s instructions (ThermoFisher Scientific, Waltham, MA). The resulting cDNA was used for gene expression analysis by qPCR using the PowerUp SYBR Green Master Mix (ThermoFisher Scientific, Waltham, MA) in 10 μL reactions with the primers listed in Table 1. Gene expression was calculated using the 2^-ΔΔCt^ formula and normalizing to the expression of the reference gene ribosomal protein L19 (RPL-19).

### Lung sections and BALF single-cell suspension

To collect Alveolar macrophages (AMs), lungs were rinsed twice with 50 mL of PBS-EDTA (ThermoFisher Scientific, Waltham, MA). Lung sections were collected using 0.8 cm biopsy punches (Integra Miltex, Princeton, NJ) and immediately placed in RPMI-1640 supplemented with 10% newborn calf serum (ThermoFisher Scientific, Waltham, MA), 2mM L-glutamine (Sigma-Aldrich, St Louis, MO), and 1% antibiotic/antimycotic (Sigma-Aldrich, St Louis, MO). Lung sections were then placed in nonculture-treated petri dishes, washed twice with PBS-EDTA, minced, and incubated for 2 hours in RPMI containing 1% antibiotic/antimycotic, 2mM L-glutamine, 2mg/mL collagenase D (Sigma-Aldrich, St Louis, MO), and 0.1 mg/mL DNase I (Sigma-Aldrich, St Louis, MO). The digestion reaction was stopped by adding 1 volume of RPMI supplemented with 10% newborn calf serum. Cells from lung sections and BALF were then passed through 70 μm cell strainers and pelleted at 300x g for 5 minutes at 4°C. Cells were resuspended in PBS-EDTA, and red blood cells were lysed with ACK lysis buffer (ThermoFisher Scientific, Waltham, MA). Following red blood cell lysis, single-cell suspensions were centrifuged at 300x g for 5 minutes at 4°C, resuspended in PBS-EDTA, and passed through 40 μm cell strainers. Cells were then counted and 1x10^6^ cells were used for flow cytometry analysis. The remaining cells were pelleted, resuspended in newborn calf serum containing 10% dimethyl sulfoxide (Sigma-Aldrich, St Louis, MO), and kept in liquid nitrogen for further analysis.

### Flow cytometry analysis

Single-cell suspensions were processed for flow cytometry as previously described by Maisonnasse, Boguyon [14] with minor modifications. Briefly, Fc cell surface receptors were blocked by incubating cells with PBS-EDTA containing 5% porcine serum (ThermoFisher Scientific, Waltham, MA) and 5% horse serum (ThermoFisher Scientific, Waltham, MA) for 30 minutes on ice. Afterward, cells were incubated for 30 minutes on ice with the following antibodies in a 1:200 dilution: Alexa 647-conjugated anti-pig SLA class II DR clone 2E9/13 (Bio-Rad, Hercules, CA), RPE-conjugated anti-pig Monocyte/Granulocyte clone 74-22-15 (Bio-Rad, Hercules, CA), and Alexa 488-conjugated anti-human CD163 clone EDHu-1 (Bio-Rad, Hercules, CA). For FLUAV detection in BALF samples, cells were incubated with the aforementioned antibodies in addition to an anti-Multi-Hemagglutinin (H3N2) polyclonal antibody in a 1:500 dilution followed by a 1-hour incubation with a PE-Alexa 610-conjugated anti-rabbit secondary antibody at a 1:500 dilution (ThermoFisher Scientific, Waltham, MA). For live/dead cell discrimination, samples were treated with LIVE/DEAD Fixable Violet Dead Cell Stain Kit (ThermoFisher Scientific, Waltham, MA) according to the manufacturer’s instructions. Finally, cells were fixed for 15 minutes with 37% formaldehyde (Sigma-Aldrich, St Louis, MO) and analyzed using a NovoCyte Quanteon flow cytometer system (Agilent, Santa Clara, CA). Data analysis was performed using FlowJo version 10.8.2 (FlowJo, Ashland, OR).

### Apoptosis determination by flow cytometry

3D4/21 cells were infected as previously described by [24]. Briefly, cells were seeded at a density of 8x10^4^ cells/cm^2^ and were incubated at 37°C under 5% CO_2_ until an 80% confluency was reached. Cells were infected with each virus at 1 MOI for 1 hour at 37°C using infection media without TPCK-treated trypsin. After infection, cells were washed three times with PBS and fresh infection media containing 250 ng/mL TPCK-treated trypsin was added. Cells were incubated at 37°C and at 12 hpi were stained using the LIVE/DEAD Fixable Violet Dead Cell Stain Kit and the Annexin V Ready Flow Conjugates for Apoptosis Detection kit (ThermoFisher Scientific, Waltham, MA) according to manufacturer’s instructions. Samples were fixed using 4% formaldehyde and analyzed as described above.

## Supporting information

S1 Fig S138 frequency among the swine 1990.4 and the 2010.1 H3 HA lineages. Amino acid frequency at position 138 (H3 numbering) among the 1990.4 and the 2010.1 lineages from isolates reported from 2009 to 2023.
(TIFF)

S2 Fig A138S does not confer a replication advantage in MDCK cells. Growth kinetics of sOH/04 (blue), hVIC/11 (yellow), and hVIC/11_A138S (red) in MDCK cells at 37 and 39°C. Experiments were performed two independent times in triplicates each time. Error bars represent the mean ± SEM.
(TIFF)

S3 Fig Affinity of H1N1 and LPAIV ΔH5N1 viruses for α2,3 and α2,6 sialylglycopolymers. Solid-phase binding assay curves of control viruses rgA/California/04/2009 (H1N1, A) with high affinity for 6’SLN (black curve) and poor biding to 3’SLN (red curve). LPAIV rgA/Vietnam/1203/04 (ΔH5N1, B) bound mostly to 3’SLN at low concentrations while poor binding to 6’SLN was detected. Experiments were performed two independent times in triplicates each time. Error bars represent the mean ± SEM.
(TIFF)

S4 Fig Oseltamivir inhibition of NA. Neuraminidase activity of hVIC/11 (A), hVIC/11^A138S^ (B), and sOH/04 (C) in presence of 0, 1, and 10 nM oseltamivir normalized to 10,000 PFU showing a dose-dependent decrease in NA activity using MUNANA as substrate and supporting the validity of the assay.
(TIFF)

S5 Fig *In vivo* swine study experimental design. Pigs were inoculated with 3 x 10^6^ TCID_50_/pig of sOH/04, or hVIC/11, or hVIC/11^A138S^. At 2 days post-infection (dpi), naïve pigs were placed in contact with inoculated pigs. After 3 days (5dpi), new contacts were introduced after removal of inoculated pigs, and this cycle was repeated for a total of 4 contacts. Pigs were euthanized at 5 dpi/6 days post contact, and bronchoalveolar lavage fluid and lung tissues were collected from seeders pigs. This illustration was created with BioRender.com.
(TIFF)

S6 Fig α2,3 and α2,6 receptors distribution in the upper and lower respiratory tract of pigs. Representative confocal images showing α2,3 (MAL II, red) and α2,6 (SNA, green) receptors distribution in the upper and lower respiratory tract. α2,6 receptors are predominant in the trachea while in the right cranial, left cranial, right caudal, left caudal, and accessory lobes both receptors are evenly distributed. Cells nuclei was stained with DAPI (blue). The scale bar represents 50 μm.
(TIFF)

S7 Fig Relative mRNA levels of pro-inflammatory cytokines, interferon-stimulated genes, and pattern-recognition receptors in the right cranial lobe. RNA was normalized to 1μg and gene expression was assessed by qPCR and normalized to RLP-19 expression in. Values are shown as log_2_ fold induction of the mean between the seeder pigs (n = 3) of each group at 5dpi. Fold induction of each group was normalized to the non-infected negative control group. Values represent the mean ± SEM. Statistical analysis was performed by two-way ANOVA. *p<0.05, **p<0.005, ***p<0.0005.
(TIFF)

S8 Fig Relative mRNA levels of pro-inflammatory cytokines, interferon-stimulated genes, and pattern-recognition receptors in the left cranial lobe. RNA was normalized to 1μg and gene expression was assessed by qPCR and normalized to RLP-19 expression in. Values are shown as log_2_ fold induction of the mean between the seeder pigs (n = 3) of each group at 5dpi. Fold induction of each group was normalized to the non-infected negative control group. Values represent the mean ± SEM. Statistical analysis was performed by two-way ANOVA. *p<0.05, **p<0.005, ***p<0.0005.
(TIFF)

S9 Fig Relative mRNA levels of pro-inflammatory cytokines, interferon-stimulated genes, and pattern-recognition receptors in the right caudal lobe. RNA was normalized to 1μg and gene expression was assessed by qPCR and normalized to RLP-19 expression in. Values are shown as log_2_ fold induction of the mean between the seeder pigs (n = 3) of each group at 5dpi. Fold induction of each group was normalized to the non-infected negative control group. Values represent the mean ± SEM. Statistical analysis was performed by two-way ANOVA. *p<0.05, **p<0.005, ***p<0.0005.
(TIFF)

S10 Fig Relative mRNA levels of pro-inflammatory cytokines, interferon-stimulated genes, and pattern-recognition receptors in the left caudal lobe. RNA was normalized to 1μg and gene expression was assessed by qPCR and normalized to RLP-19 expression in. Values are shown as log_2_ fold induction of the mean between the seeder pigs (n = 3) of each group at 5dpi. Fold induction of each group was normalized to the non-infected negative control group. Values represent the mean ± SEM. Statistical analysis was performed by two-way ANOVA. *p<0.05, **p<0.005, ***p<0.0005.
(TIFF)

S11 Fig Relative mRNA levels of pro-inflammatory cytokines, interferon-stimulated genes, and pattern-recognition receptors in the accessory lobe. RNA was normalized to 1μg and gene expression was assessed by qPCR and normalized to RLP-19 expression in. Values are shown as log_2_ fold induction of the mean between the seeder pigs (n = 3) of each group at 5dpi. Fold induction of each group was normalized to the non-infected negative control group. Values represent the mean ± SEM. Statistical analysis was performed by two-way ANOVA. *p<0.05, **p<0.005, ***p<0.0005.
(TIFF)

S12 Fig Flow cytometry analysis of lung and BALF samples. Representative flow cytometry results showing APC (top panels) and cDC1, cDC2, moDC, moMϕ, and PiAMs (bottom panels) in right cranial (A), left cranial (B), right caudal (C), left caudal (D), and accessory lobes (E) from mock-, hVIC/11-, hVIC/11^A138S^-, and sOH/04-infected pigs. The gating strategy performed in tissue samples was also used in BALF (F) samples and only one population gated from MHCII^high^ CD163^pos^ cells appeared.
(TIFF)

## References

[1] Ito T, Kawaoka Y. Host-range barrier of influenza A viruses. Vet Microbiol. 2000;74(1–2):71–5. doi: 10.1016/s0378-1135(00)00167-x . 10799779

[2] Bourret V. Avian influenza viruses in pigs: An overview. Vet J. 2018;239:7–14. Epub 20180719. doi: 10.1016/j.tvjl.2018.07.005 . 30197112

[3] Smith GJ, Vijaykrishna D, Bahl J, Lycett SJ, Worobey M, Pybus OG, et al. Origins and evolutionary genomics of the 2009 swine-origin H1N1 influenza A epidemic. Nature. 2009;459(7250):1122–5. doi: 10.1038/nature08182 . 19516283

[4] Nelson MI, Wentworth DE, Culhane MR, Vincent AL, Viboud C, LaPointe MP, et al. Introductions and evolution of human-origin seasonal influenza a viruses in multinational swine populations. J Virol. 2014;88(17):10110–9. Epub 20140625. doi: 10.1128/JVI.01080-14 ; PubMed Central PMCID: PMC4136342. 24965467 PMC4136342

[5] Rajao DS, Vincent AL, Perez DR. Adaptation of Human Influenza Viruses to Swine. Front Vet Sci. 2018;5:347. Epub 20190122. doi: 10.3389/fvets.2018.00347 ; PubMed Central PMCID: PMC6349779. 30723723 PMC6349779

[6] Anderson TK, Chang J, Arendsee ZW, Venkatesh D, Souza CK, Kimble JB, et al. Swine Influenza A Viruses and the Tangled Relationship with Humans. Cold Spring Harb Perspect Med. 2021;11(3). Epub 20210301. doi: 10.1101/cshperspect.a038737 ; PubMed Central PMCID: PMC7919397. 31988203 PMC7919397

[7] Rajao DS, Gauger PC, Anderson TK, Lewis NS, Abente EJ, Killian ML, et al. Novel Reassortant Human-Like H3N2 and H3N1 Influenza A Viruses Detected in Pigs Are Virulent and Antigenically Distinct from Swine Viruses Endemic to the United States. J Virol. 2015;89(22):11213–22. Epub 20150826. doi: 10.1128/JVI.01675-15 ; PubMed Central PMCID: PMC4645639. 26311895 PMC4645639

[8] Rajao DS, Abente EJ, Powell JD, Bolton MJ, Gauger PC, Arruda B, et al. Changes in the Hemagglutinin and Internal Gene Segments Were Needed for Human Seasonal H3 Influenza A Virus to Efficiently Infect and Replicate in Swine. Pathogens. 2022;11(9). Epub 20220825. doi: 10.3390/pathogens11090967 ; PubMed Central PMCID: PMC9501159. 36145399 PMC9501159

[9] Neumann G, Kawaoka Y. Host range restriction and pathogenicity in the context of influenza pandemic. Emerg Infect Dis. 2006;12(6):881–6. doi: 10.3201/eid1206.051336 ; PubMed Central PMCID: PMC3373033. 16707041 PMC3373033

[10] Subbarao EK, London W, Murphy BR. A single amino acid in the PB2 gene of influenza A virus is a determinant of host range. J Virol. 1993;67(4):1761–4. doi: 10.1128/JVI.67.4.1761-1764.1993 ; PubMed Central PMCID: PMC240216. 8445709 PMC240216

[11] Connor RJ, Kawaoka Y, Webster RG, Paulson JC. Receptor specificity in human, avian, and equine H2 and H3 influenza virus isolates. Virology. 1994;205(1):17–23. doi: 10.1006/viro.1994.1615 . 7975212

[12] Stevens J, Blixt O, Glaser L, Taubenberger JK, Palese P, Paulson JC, et al. Glycan microarray analysis of the hemagglutinins from modern and pandemic influenza viruses reveals different receptor specificities. J Mol Biol. 2006;355(5):1143–55. Epub 20051118. doi: 10.1016/j.jmb.2005.11.002 . 16343533

[13] Xu R, Zhu X, McBride R, Nycholat CM, Yu W, Paulson JC, et al. Functional balance of the hemagglutinin and neuraminidase activities accompanies the emergence of the 2009 H1N1 influenza pandemic. J Virol. 2012;86(17):9221–32. Epub 20120620. doi: 10.1128/JVI.00697-12 ; PubMed Central PMCID: PMC3416152. 22718832 PMC3416152

[14] Maisonnasse P, Bouguyon E, Piton G, Ezquerra A, Urien C, Deloizy C, et al. The respiratory DC/macrophage network at steady-state and upon influenza infection in the swine biomedical model. Mucosal Immunol. 2016;9(4):835–49. Epub 20151104. doi: 10.1038/mi.2015.105 . 26530136

[15] Kumagai Y, Takeuchi O, Kato H, Kumar H, Matsui K, Morii E, et al. Alveolar macrophages are the primary interferon-alpha producer in pulmonary infection with RNA viruses. Immunity. 2007;27(2):240–52. doi: 10.1016/j.immuni.2007.07.013 . 17723216

[16] Wang J, Nikrad MP, Travanty EA, Zhou B, Phang T, Gao B, et al. Innate immune response of human alveolar macrophages during influenza A infection. PLoS One. 2012;7(3):e29879. Epub 20120302. doi: 10.1371/journal.pone.0029879 ; PubMed Central PMCID: PMC3292548. 22396727 PMC3292548

[17] Wijburg OL, DiNatale S, Vadolas J, van Rooijen N, Strugnell RA. Alveolar macrophages regulate the induction of primary cytotoxic T-lymphocyte responses during influenza virus infection. J Virol. 1997;71(12):9450–7. doi: 10.1128/JVI.71.12.9450-9457.1997 ; PubMed Central PMCID: PMC230250. 9371606 PMC230250

[18] He W, Chen CJ, Mullarkey CE, Hamilton JR, Wong CK, Leon PE, et al. Alveolar macrophages are critical for broadly-reactive antibody-mediated protection against influenza A virus in mice. Nat Commun. 2017;8(1):846. Epub 20171010. doi: 10.1038/s41467-017-00928-3 ; PubMed Central PMCID: PMC5635038. 29018261 PMC5635038

[19] Kim HM, Kang YM, Ku KB, Park EH, Yum J, Kim JC, et al. The severe pathogenicity of alveolar macrophage-depleted ferrets infected with 2009 pandemic H1N1 influenza virus. Virology. 2013;444(1–2):394–403. Epub 20130723. doi: 10.1016/j.virol.2013.07.006 . 23890814

[20] Tate MD, Pickett DL, van Rooijen N, Brooks AG, Reading PC. Critical role of airway macrophages in modulating disease severity during influenza virus infection of mice. J Virol. 2010;84(15):7569–80. Epub 20100526. doi: 10.1128/JVI.00291-10 ; PubMed Central PMCID: PMC2897615. 20504924 PMC2897615

[21] Wein AN, Dunbar PR, McMaster SR, Li ZT, Denning TL, Kohlmeier JE. IL-36gamma Protects against Severe Influenza Infection by Promoting Lung Alveolar Macrophage Survival and Limiting Viral Replication. J Immunol. 2018;201(2):573–82. Epub 20180530. doi: 10.4049/jimmunol.1701796 ; PubMed Central PMCID: PMC6089355. 29848754 PMC6089355

[22] van Riel D, Leijten LM, van der Eerden M, Hoogsteden HC, Boven LA, Lambrecht BN, et al. Highly pathogenic avian influenza virus H5N1 infects alveolar macrophages without virus production or excessive TNF-alpha induction. PLoS Pathog. 2011;7(6):e1002099. Epub 20110623. doi: 10.1371/journal.ppat.1002099 ; PubMed Central PMCID: PMC3121882. 21731493 PMC3121882

[23] Yu WC, Chan RW, Wang J, Travanty EA, Nicholls JM, Peiris JS, et al. Viral replication and innate host responses in primary human alveolar epithelial cells and alveolar macrophages infected with influenza H5N1 and H1N1 viruses. J Virol. 2011;85(14):6844–55. Epub 20110504. doi: 10.1128/JVI.02200-10 ; PubMed Central PMCID: PMC3126566. 21543489 PMC3126566

[24] Chang P, Kuchipudi SV, Mellits KH, Sebastian S, James J, Liu J, et al. Early apoptosis of porcine alveolar macrophages limits avian influenza virus replication and pro-inflammatory dysregulation. Sci Rep. 2015;5:17999. Epub 20151208. doi: 10.1038/srep17999 ; PubMed Central PMCID: PMC4672291. 26642934 PMC4672291

[25] Perrone LA, Plowden JK, Garcia-Sastre A, Katz JM, Tumpey TM. H5N1 and 1918 pandemic influenza virus infection results in early and excessive infiltration of macrophages and neutrophils in the lungs of mice. PLoS Pathog. 2008;4(8):e1000115. Epub 20080801. doi: 10.1371/journal.ppat.1000115 ; PubMed Central PMCID: PMC2483250. 18670648 PMC2483250

[26] Zhang J, Miao J, Hou J, Lu C. The effects of H3N2 swine influenza virus infection on TLRs and RLRs signaling pathways in porcine alveolar macrophages. Virol J. 2015;12:61. Epub 20150414. doi: 10.1186/s12985-015-0284-6 ; PubMed Central PMCID: PMC4487856. 26021751 PMC4487856

[27] Tate MD, Schilter HC, Brooks AG, Reading PC. Responses of mouse airway epithelial cells and alveolar macrophages to virulent and avirulent strains of influenza A virus. Viral Immunol. 2011;24(2):77–88. doi: 10.1089/vim.2010.0118 . 21449718

[28] Huang S, Zhu B, Cheon IS, Goplen NP, Jiang L, Zhang R, et al. PPAR-gamma in Macrophages Limits Pulmonary Inflammation and Promotes Host Recovery following Respiratory Viral Infection. J Virol. 2019;93(9). Epub 20190417. doi: 10.1128/JVI.00030-19 ; PubMed Central PMCID: PMC6475778. 30787149 PMC6475778

[29] Gopalakrishnan A, Joseph J, Shirey KA, Keegan AD, Boukhvalova MS, Vogel SN, et al. Protection against influenza-induced Acute Lung Injury (ALI) by enhanced induction of M2a macrophages: possible role of PPARgamma/RXR ligands in IL-4-induced M2a macrophage differentiation. Front Immunol. 2022;13:968336. Epub 20220816. doi: 10.3389/fimmu.2022.968336 ; PubMed Central PMCID: PMC9424652. 36052067 PMC9424652

[30] Schneider C, Nobs SP, Kurrer M, Rehrauer H, Thiele C, Kopf M. Induction of the nuclear receptor PPAR-gamma by the cytokine GM-CSF is critical for the differentiation of fetal monocytes into alveolar macrophages. Nat Immunol. 2014;15(11):1026–37. Epub 20140928. doi: 10.1038/ni.3005 . 25263125

[31] Ditiatkovski M, Toh BH, Bobik A. GM-CSF deficiency reduces macrophage PPAR-gamma expression and aggravates atherosclerosis in ApoE-deficient mice. Arterioscler Thromb Vasc Biol. 2006;26(10):2337–44. Epub 20060727. doi: 10.1161/01.ATV.0000238357.60338.90 . 16873730

[32] Guilliams M, De Kleer I, Henri S, Post S, Vanhoutte L, De Prijck S, et al. Alveolar macrophages develop from fetal monocytes that differentiate into long-lived cells in the first week of life via GM-CSF. J Exp Med. 2013;210(10):1977–92. Epub 20130916. doi: 10.1084/jem.20131199 ; PubMed Central PMCID: PMC3782041. 24043763 PMC3782041

[33] Joshi N, Walter JM, Misharin AV. Alveolar Macrophages. Cell Immunol. 2018;330:86–90. Epub 20180120. doi: 10.1016/j.cellimm.2018.01.005 . 29370889

[34] Cline TD, Beck D, Bianchini E. Influenza virus replication in macrophages: balancing protection and pathogenesis. J Gen Virol. 2017;98(10):2401–12. Epub 20170908. doi: 10.1099/jgv.0.000922 ; PubMed Central PMCID: PMC5725990. 28884667 PMC5725990

[35] Mo JS, Abente EJ, Cardenas Perez M, Sutton TC, Cowan B, Ferreri LM, et al. Transmission of Human Influenza A Virus in Pigs Selects for Adaptive Mutations on the HA Gene. J Virol. 2022;96(22):e0148022. Epub 20221101. doi: 10.1128/jvi.01480-22 ; PubMed Central PMCID: PMC9682980. 36317880 PMC9682980

[36] Busch MG, Bateman AC, Landolt GA, Karasin AI, Brockman-Schneider RA, Gern JE, et al. Identification of amino acids in the HA of H3 influenza viruses that determine infectivity levels in primary swine respiratory epithelial cells. Virus Res. 2008;133(2):269–79. Epub 20080310. doi: 10.1016/j.virusres.2008.01.014 . 18329747

[37] Obadan AO, Santos J, Ferreri L, Thompson AJ, Carnaccini S, Geiger G, et al. Flexibility In Vitro of Amino Acid 226 in the Receptor-Binding Site of an H9 Subtype Influenza A Virus and Its Effect In Vivo on Virus Replication, Tropism, and Transmission. J Virol. 2019;93(6). Epub 20190305. doi: 10.1128/JVI.02011-18 ; PubMed Central PMCID: PMC6401463. 30567980 PMC6401463

[38] Santos JJS, Abente EJ, Obadan AO, Thompson AJ, Ferreri L, Geiger G, et al. Plasticity of Amino Acid Residue 145 Near the Receptor Binding Site of H3 Swine Influenza A Viruses and Its Impact on Receptor Binding and Antibody Recognition. J Virol. 2019;93(2). Epub 20190104. doi: 10.1128/JVI.01413-18 ; PubMed Central PMCID: PMC6321904. 30355680 PMC6321904

[39] Van Poucke SG, Nicholls JM, Nauwynck HJ, Van Reeth K. Replication of avian, human and swine influenza viruses in porcine respiratory explants and association with sialic acid distribution. Virol J. 2010;7:38. Epub 20100216. doi: 10.1186/1743-422X-7-38 ; PubMed Central PMCID: PMC2829537. 20158900 PMC2829537

[40] Rodriguez RM, Suarez-Alvarez B, Lavin JL, Ascension AM, Gonzalez M, Lozano JJ, et al. Signal Integration and Transcriptional Regulation of the Inflammatory Response Mediated by the GM-/M-CSF Signaling Axis in Human Monocytes. Cell Rep. 2019;29(4):860–72 e5. doi: 10.1016/j.celrep.2019.09.035 . 31644909

[41] Gao S, Anderson TK, Walia RR, Dorman KS, Janas-Martindale A, Vincent AL. The genomic evolution of H1 influenza A viruses from swine detected in the United States between 2009 and 2016. J Gen Virol. 2017;98(8):2001–10. Epub 20170731. doi: 10.1099/jgv.0.000885 ; PubMed Central PMCID: PMC5817270. 28758634 PMC5817270

[42] Nelson MI, Gramer MR, Vincent AL, Holmes EC. Global transmission of influenza viruses from humans to swine. J Gen Virol. 2012;93(Pt 10):2195–203. Epub 20120712. doi: 10.1099/vir.0.044974-0 ; PubMed Central PMCID: PMC3541789. 22791604 PMC3541789

[43] Nelson MI, Stratton J, Killian ML, Janas-Martindale A, Vincent AL. Continual Reintroduction of Human Pandemic H1N1 Influenza A Viruses into Swine in the United States, 2009 to 2014. J Virol. 2015;89(12):6218–26. Epub 20150401. doi: 10.1128/JVI.00459-15 ; PubMed Central PMCID: PMC4474294. 25833052 PMC4474294

[44] Liang H, Lam TT, Fan X, Chen X, Zeng Y, Zhou J, et al. Expansion of genotypic diversity and establishment of 2009 H1N1 pandemic-origin internal genes in pigs in China. J Virol. 2014;88(18):10864–74. Epub 20140709. doi: 10.1128/JVI.01327-14 ; PubMed Central PMCID: PMC4178866. 25008935 PMC4178866

[45] Rajao DS, Walia RR, Campbell B, Gauger PC, Janas-Martindale A, Killian ML, et al. Reassortment between Swine H3N2 and 2009 Pandemic H1N1 in the United States Resulted in Influenza A Viruses with Diverse Genetic Constellations with Variable Virulence in Pigs. J Virol. 2017;91(4). Epub 20170131. doi: 10.1128/JVI.01763-16 ; PubMed Central PMCID: PMC5286888. 27928015 PMC5286888

[46] Ngo LT, Hiromoto Y, Pham VP, Le HT, Nguyen HT, Le VT, et al. Isolation of novel triple-reassortant swine H3N2 influenza viruses possessing the hemagglutinin and neuraminidase genes of a seasonal influenza virus in Vietnam in 2010. Influenza Other Respir Viruses. 2012;6(1):6–10. Epub 20110613. doi: 10.1111/j.1750-2659.2011.00267.x ; PubMed Central PMCID: PMC4941553. 21668659 PMC4941553

[47] Kaplan BS, Anderson TK, Chang J, Santos J, Perez D, Lewis N, et al. Evolution and Antigenic Advancement of N2 Neuraminidase of Swine Influenza A Viruses Circulating in the United States following Two Separate Introductions from Human Seasonal Viruses. J Virol. 2021;95(20):e0063221. Epub 20210811. doi: 10.1128/JVI.00632-21 ; PubMed Central PMCID: PMC8475526. 34379513 PMC8475526

[48] Karasinski J, White L, Zhang Y, Wang E, Andreescu S, Sadik OA, et al. Detection and identification of bacteria using antibiotic susceptibility and a multi-array electrochemical sensor with pattern recognition. Biosens Bioelectron. 2007;22(11):2643–9. Epub 20061212. doi: 10.1016/j.bios.2006.10.037 . 17169547

[49] Vijaykrishna D, Smith GJ, Pybus OG, Zhu H, Bhatt S, Poon LL, et al. Long-term evolution and transmission dynamics of swine influenza A virus. Nature. 2011;473(7348):519–22. doi: 10.1038/nature10004 . 21614079

[50] Zhang Y, Zhao C, Hou Y, Chen Y, Meng F, Zhuang Y, et al. Pandemic threat posed by H3N2 avian influenza virus. Sci China Life Sci. 2021;64(11):1984–7. Epub 20210322. doi: 10.1007/s11427-021-1916-4 . 33765225

[51] Zanin M, Marathe B, Wong SS, Yoon SW, Collin E, Oshansky C, et al. Pandemic Swine H1N1 Influenza Viruses with Almost Undetectable Neuraminidase Activity Are Not Transmitted via Aerosols in Ferrets and Are Inhibited by Human Mucus but Not Swine Mucus. J Virol. 2015;89(11):5935–48. Epub 20150325. doi: 10.1128/JVI.02537-14 ; PubMed Central PMCID: PMC4442420. 25810540 PMC4442420

[52] Wan H, Sorrell EM, Song H, Hossain MJ, Ramirez-Nieto G, Monne I, et al. Replication and transmission of H9N2 influenza viruses in ferrets: evaluation of pandemic potential. PLoS One. 2008;3(8):e2923. Epub 20080813. doi: 10.1371/journal.pone.0002923 ; PubMed Central PMCID: PMC2500216. 18698430 PMC2500216

[53] Belser JA, Gustin KM, Pearce MB, Maines TR, Zeng H, Pappas C, et al. Pathogenesis and transmission of avian influenza A (H7N9) virus in ferrets and mice. Nature. 2013;501(7468):556–9. Epub 20130710. doi: 10.1038/nature12391 ; PubMed Central PMCID: PMC7094885. 23842497 PMC7094885

[54] Pearce MB, Jayaraman A, Pappas C, Belser JA, Zeng H, Gustin KM, et al. Pathogenesis and transmission of swine origin A(H3N2)v influenza viruses in ferrets. Proc Natl Acad Sci U S A. 2012;109(10):3944–9. Epub 20120221. doi: 10.1073/pnas.1119945109 ; PubMed Central PMCID: PMC3309732. 22355116 PMC3309732

[55] Pascua PN, Song MS, Lee JH, Baek YH, Kwon HI, Park SJ, et al. Virulence and transmissibility of H1N2 influenza virus in ferrets imply the continuing threat of triple-reassortant swine viruses. Proc Natl Acad Sci U S A. 2012;109(39):15900–5. Epub 20120910. doi: 10.1073/pnas.1205576109 ; PubMed Central PMCID: PMC3465388. 23019374 PMC3465388

[56] Xie C, Su W, Sia SF, Choy KT, Morrell S, Zhou J, et al. A(H1N1)pdm09 Influenza Viruses Replicating in Ferret Upper or Lower Respiratory Tract Differed in Onward Transmission Potential by Air. J Infect Dis. 2022;225(1):65–74. doi: 10.1093/infdis/jiab286 ; PubMed Central PMCID: PMC8730494. 34036370 PMC8730494

[57] Seo SH, Webster RG. Tumor Necrosis Factor Alpha Exerts Powerful Anti-Influenza Virus Effects in Lung Epithelial Cells. Journal of Virology. 2002;76(3):1071–6. doi: 10.1128/jvi.76.3.1071-1076.2002 11773383 PMC135862

[58] Svitek N, Rudd PA, Obojes K, Pillet S, von Messling V. Severe seasonal influenza in ferrets correlates with reduced interferon and increased IL-6 induction. Virology. 2008;376(1):53–9. doi: 10.1016/j.virol.2008.02.035 . 18420248

[59] Khatri M, Dwivedi V, Krakowka S, Manickam C, Ali A, Wang L, et al. Swine influenza H1N1 virus induces acute inflammatory immune responses in pig lungs: a potential animal model for human H1N1 influenza virus. J Virol. 2010;84(21):11210–8. Epub 2010/08/20. doi: 10.1128/JVI.01211-10 ; PubMed Central PMCID: PMC2953174. 20719941 PMC2953174

[60] Barbe F, Atanasova K, Van Reeth K. Cytokines and acute phase proteins associated with acute swine influenza infection in pigs. Vet J. 2011;187(1):48–53. Epub 20100122. doi: 10.1016/j.tvjl.2009.12.012 ; PubMed Central PMCID: PMC7129392. 20097110 PMC7129392

[61] Vandoorn E, Stadejek W, Parys A, Chepkwony S, Chiers K, Van Reeth K. Pathobiology of an NS1-Truncated H3N2 Swine Influenza Virus Strain in Pigs. J Virol. 2022;96(11):e0051922. Epub 20220512. doi: 10.1128/jvi.00519-22 ; PubMed Central PMCID: PMC9175629. 35546120 PMC9175629

[62] Rot A. Neutrophil attractant/activation protein-1 (interleukin-8) induces in vitro neutrophil migration by haptotactic mechanism. Eur J Immunol. 1993;23(1):303–6. doi: 10.1002/eji.1830230150 . 8419183

[63] Lam WY, Yeung AC, Chu IM, Chan PK. Profiles of cytokine and chemokine gene expression in human pulmonary epithelial cells induced by human and avian influenza viruses. Virol J. 2010;7:344. Epub 20101126. doi: 10.1186/1743-422X-7-344 ; PubMed Central PMCID: PMC3002310. 21108843 PMC3002310

[64] Veldhuis WB, Derksen JW, Floris S, Van Der Meide PH, De Vries HE, Schepers J, et al. Interferon-beta blocks infiltration of inflammatory cells and reduces infarct volume after ischemic stroke in the rat. J Cereb Blood Flow Metab. 2003;23(9):1029–39. doi: 10.1097/01.WCB.0000080703.47016.B6 . 12973019

[65] Cobelens PM, Tiebosch IA, Dijkhuizen RM, van der Meide PH, Zwartbol R, Heijnen CJ, et al. Interferon-beta attenuates lung inflammation following experimental subarachnoid hemorrhage. Crit Care. 2010;14(4):R157. Epub 20100823. doi: 10.1186/cc9232 ; PubMed Central PMCID: PMC2945141. 20731855 PMC2945141

[66] Ghoneim HE, Thomas PG, McCullers JA. Depletion of alveolar macrophages during influenza infection facilitates bacterial superinfections. J Immunol. 2013;191(3):1250–9. Epub 20130626. doi: 10.4049/jimmunol.1300014 ; PubMed Central PMCID: PMC4907362. 23804714 PMC4907362

[67] Seo SH, Webby R, Webster RG. No apoptotic deaths and different levels of inductions of inflammatory cytokines in alveolar macrophages infected with influenza viruses. Virology. 2004;329(2):270–9. doi: 10.1016/j.virol.2004.08.019 . 15518807

[68] Dai CH, Gao ZC, Cheng JH, Yang L, Wu ZC, Wu SL, et al. The Competitive Endogenous RNA (ceRNA) Regulation in Porcine Alveolar Macrophages (3D4/21) Infected by Swine Influenza Virus (H1N1 and H3N2). Int J Mol Sci. 2022;23(3). Epub 20220207. doi: 10.3390/ijms23031875 ; PubMed Central PMCID: PMC8836399. 35163797 PMC8836399

[69] Ettensohn DB, Frampton MW, Nichols JE, Roberts NJ Jr. Human Alveolar Macrophages May Not Be Susceptible to Direct Infection by a Human Influenza Virus. J Infect Dis. 2016;214(11):1658–65. Epub 20160906. doi: 10.1093/infdis/jiw413 ; PubMed Central PMCID: PMC5144727. 27601618 PMC5144727

[70] Reddy RC. Immunomodulatory role of PPAR-gamma in alveolar macrophages. J Investig Med. 2008;56(2):522–7. doi: 10.2310/JIM.0b013e3181659972 . 18317435

[71] Zhang H, Alford T, Liu S, Zhou D, Wang J. Influenza virus causes lung immunopathology through down-regulating PPARgamma activity in macrophages. Front Immunol. 2022;13:958801. Epub 20220825. doi: 10.3389/fimmu.2022.958801 ; PubMed Central PMCID: PMC9452838. 36091002 PMC9452838

[72] Huang S, Jiang L, Cheon IS, Sun J. Targeting Peroxisome Proliferator-Activated Receptor-Gamma Decreases Host Mortality After Influenza Infection in Obese Mice. Viral Immunol. 2019;32(4):161–9. Epub 20190419. doi: 10.1089/vim.2019.0016 ; PubMed Central PMCID: PMC6534095. 31009317 PMC6534095

[73] Wakao H, Wakao R, Oda A, Fujita H. Constitutively active Stat5A and Stat5B promote adipogenesis. Environ Health Prev Med. 2011;16(4):247–52. Epub 20101201. doi: 10.1007/s12199-010-0193-7 ; PubMed Central PMCID: PMC3117211. 21431790 PMC3117211

[74] Yang H, Dong Y, Bian Y, Xu N, Wu Y, Yang F, et al. The influenza virus PB2 protein evades antiviral innate immunity by inhibiting JAK1/STAT signalling. Nat Commun. 2022;13(1):6288. Epub 20221021. doi: 10.1038/s41467-022-33909-2 ; PubMed Central PMCID: PMC9586965. 36271046 PMC9586965

[75] Uetani K, Hiroi M, Meguro T, Ogawa H, Kamisako T, Ohmori Y, et al. Influenza A virus abrogates IFN-gamma response in respiratory epithelial cells by disruption of the Jak/Stat pathway. Eur J Immunol. 2008;38(6):1559–73. doi: 10.1002/eji.200737045 . 18493979

[76] Feng M, Zhang Q, Wu W, Chen L, Gu S, Ye Y, et al. Inducible Guanylate-Binding Protein 7 Facilitates Influenza A Virus Replication by Suppressing Innate Immunity via NF-kappaB and JAK-STAT Signaling Pathways. J Virol. 2021;95(6). Epub 20210224. doi: 10.1128/JVI.02038-20 ; PubMed Central PMCID: PMC8094947. 33408175 PMC8094947

[77] Fleming SB. Viral Inhibition of the IFN-Induced JAK/STAT Signalling Pathway: Development of Live Attenuated Vaccines by Mutation of Viral-Encoded IFN-Antagonists. Vaccines (Basel). 2016;4(3). Epub 20160629. doi: 10.3390/vaccines4030023 ; PubMed Central PMCID: PMC5041017. 27367734 PMC5041017

[78] REED LJ, MUENCH H. A SIMPLE METHOD OF ESTIMATING FIFTY PER CENT ENDPOINTS12. American Journal of Epidemiology. 1938;27(3):493–7. doi: 10.1093/oxfordjournals.aje.a118408

[79] Marathe BM, Leveque V, Klumpp K, Webster RG, Govorkova EA. Determination of neuraminidase kinetic constants using whole influenza virus preparations and correction for spectroscopic interference by a fluorogenic substrate. PLoS One. 2013;8(8):e71401. Epub 20130815. doi: 10.1371/journal.pone.0071401 ; PubMed Central PMCID: PMC3744557. 23977037 PMC3744557

[80] Matrosovich MN, Gambaryan AS. Solid-phase assays of receptor-binding specificity. Methods Mol Biol. 2012;865:71–94. doi: 10.1007/978-1-61779-621-0_5 . 22528154

[81] Zaqout S, Becker LL, Kaindl AM. Immunofluorescence Staining of Paraffin Sections Step by Step. Front Neuroanat. 2020;14:582218. Epub 20201109. doi: 10.3389/fnana.2020.582218 ; PubMed Central PMCID: PMC7680859. 33240048 PMC7680859

[82] Delgado-Ortega M, Melo S, Punyadarsaniya D, Rame C, Olivier M, Soubieux D, et al. Innate immune response to a H3N2 subtype swine influenza virus in newborn porcine trachea cells, alveolar macrophages, and precision-cut lung slices. Vet Res. 2014;45(1):42. Epub 20140409. doi: 10.1186/1297-9716-45-42 ; PubMed Central PMCID: PMC4021251. 24712747 PMC4021251

[83] Paulin SM, Jagannathan A, Campbell J, Wallis TS, Stevens MP. Net replication of Salmonella enterica serovars Typhimurium and Choleraesuis in porcine intestinal mucosa and nodes is associated with their differential virulence. Infect Immun. 2007;75(8):3950–60. Epub 20070604. doi: 10.1128/IAI.00366-07 ; PubMed Central PMCID: PMC1952012. 17548482 PMC1952012

[84] Cui J, Chen W, Liu J, Xu T, Zeng Y. Study on quantitative expression of PPARgamma and ADRP in muscle and its association with intramuscular fat deposition of pig. Springerplus. 2016;5(1):1501. Epub 20160907. doi: 10.1186/s40064-016-3187-0 ; PubMed Central PMCID: PMC5014771. 27652074 PMC5014771

[85] Yang ZZ, Habib M, Shuai JB, Fang WH. Detection of PCV2 DNA by SYBR Green I-based quantitative PCR. J Zhejiang Univ Sci B. 2007;8(3):162–9. doi: 10.1631/jzus.2007.B0162 ; PubMed Central PMCID: PMC1810386. 17323427 PMC1810386

[86] Zheng LL, Chai LY, Tian RB, Zhao Y, Chen HY, Wang ZY. Simultaneous detection of porcine reproductive and respiratory syndrome virus and porcine circovirus 3 by SYBR Green capital I, Ukrainian-based duplex real-time PCR. Mol Cell Probes. 2020;49:101474. Epub 20191023. doi: 10.1016/j.mcp.2019.101474 . 31655106

[87] Vranckx K, Maes D, Marchioro SB, Villarreal I, Chiers K, Pasmans F, et al. Vaccination reduces macrophage infiltration in bronchus-associated lymphoid tissue in pigs infected with a highly virulent Mycoplasma hyopneumoniae strain. BMC Vet Res. 2012;8:24. Epub 20120312. doi: 10.1186/1746-6148-8-24 ; PubMed Central PMCID: PMC3349615. 22409839 PMC3349615

