# Supplementary figures and images for: Amino acid 138 in the HA of a H3N2 subtype influenza A virus increases affinity for the lower respiratory tract and alveolar macrophages in pigs
Source: PLoS Pathog. 2024 Feb 20;20(2):e1012026. doi: 10.1371/journal.ppat.1012026 (PMC10906893; doi:10.1371/journal.ppat.1012026)

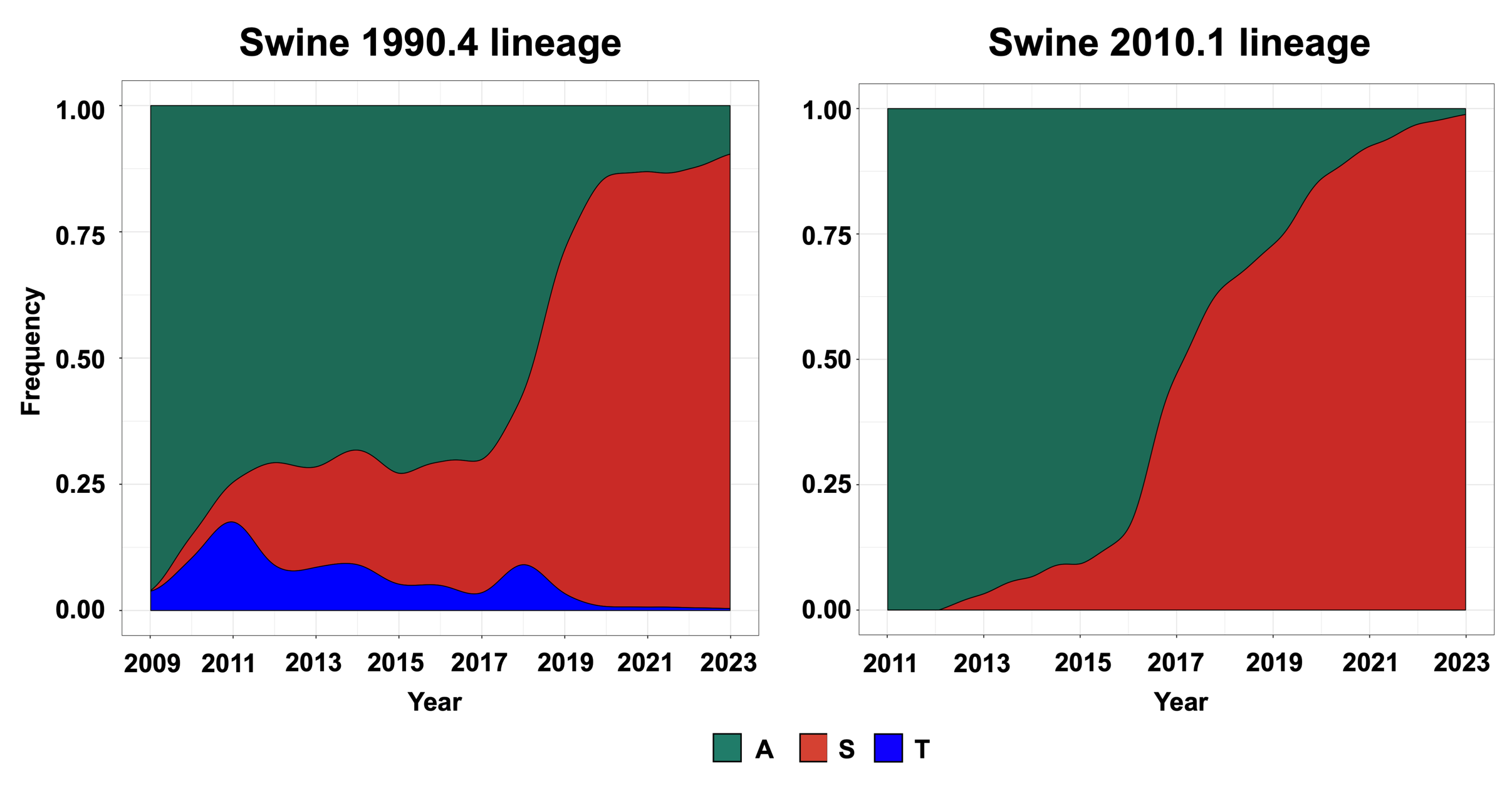

Supplement: S1 Fig — Amino acid frequency at position 138 (H3 numbering) among the 1990.4 and the 2010.1 lineages from isolates reported from 2009 to 2023. (TIFF) [file ppat.1012026.s001.tiff]

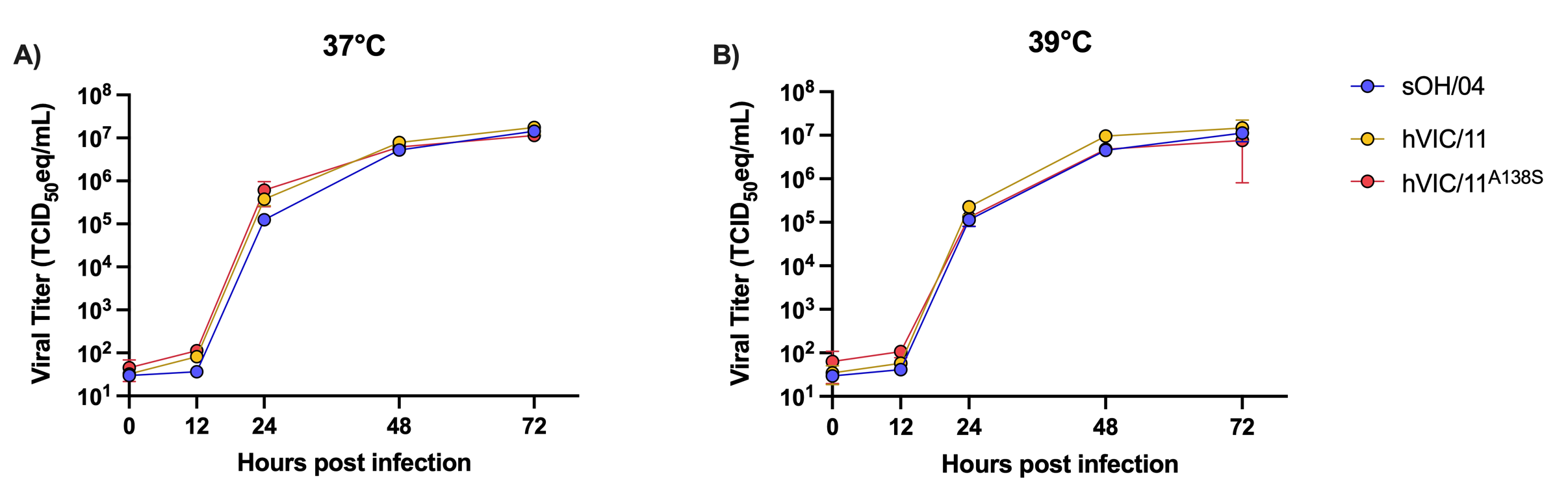

Supplement: S2 Fig — Growth kinetics of sOH/04 (blue), hVIC/11 (yellow), and hVIC/11_A138S (red) in MDCK cells at 37 and 39°C. Experiments were performed two independent times in triplicates each time. Error bars represent the mean ± SEM. (TIFF) [file ppat.1012026.s002.tiff]

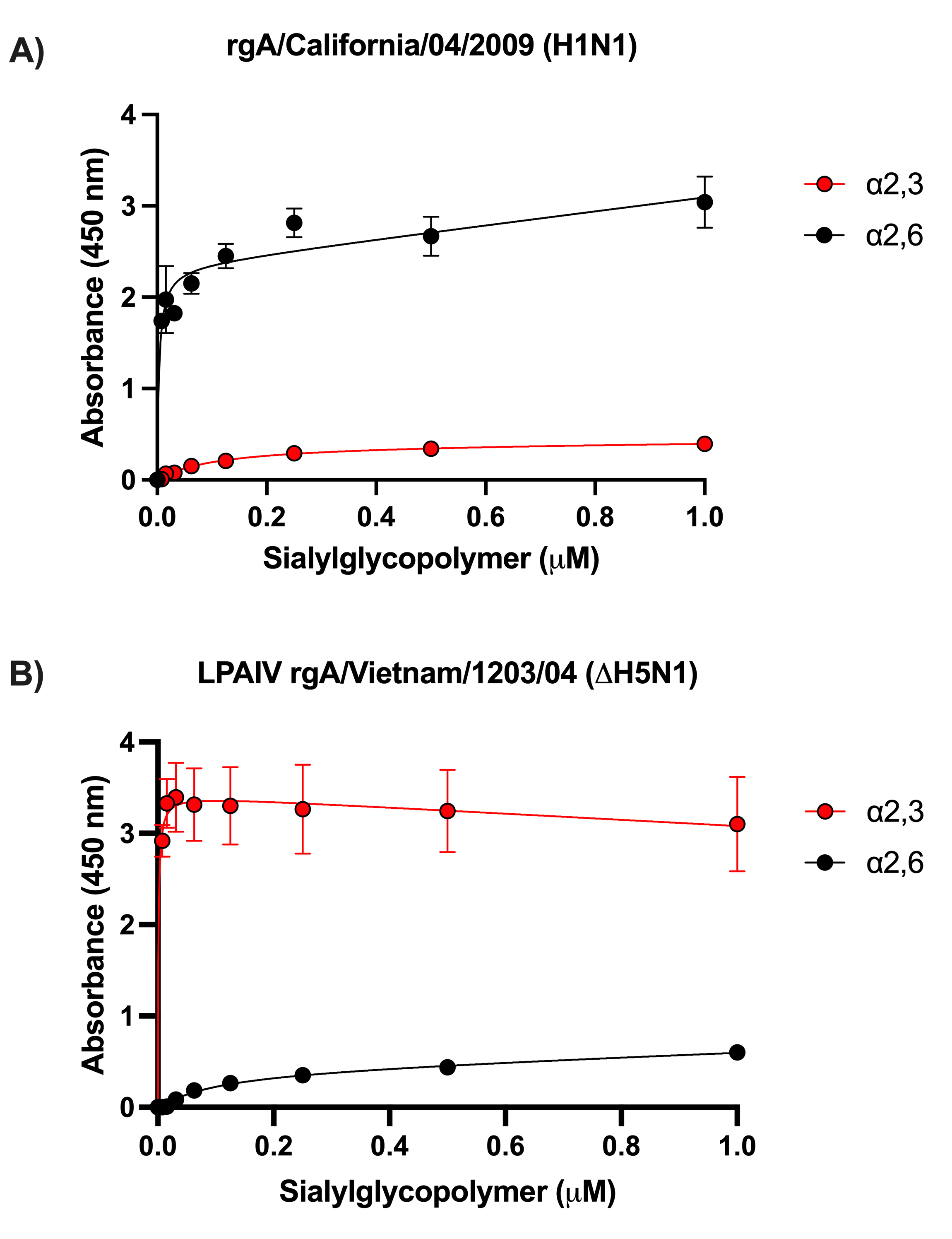

Supplement: S3 Fig — Solid-phase binding assay curves of control viruses rgA/California/04/2009 (H1N1, A) with high affinity for 6’SLN (black curve) and poor biding to 3’SLN (red curve). LPAIV rgA/Vietnam/1203/04 (ΔH5N1, B) bound mostly to 3’SLN at low concentrations while poor binding to 6’SLN was detected. Experiments were performed two independent times in triplicates each time. Error bars represent the mean ± SEM. (TIFF) [file ppat.1012026.s003.tiff]

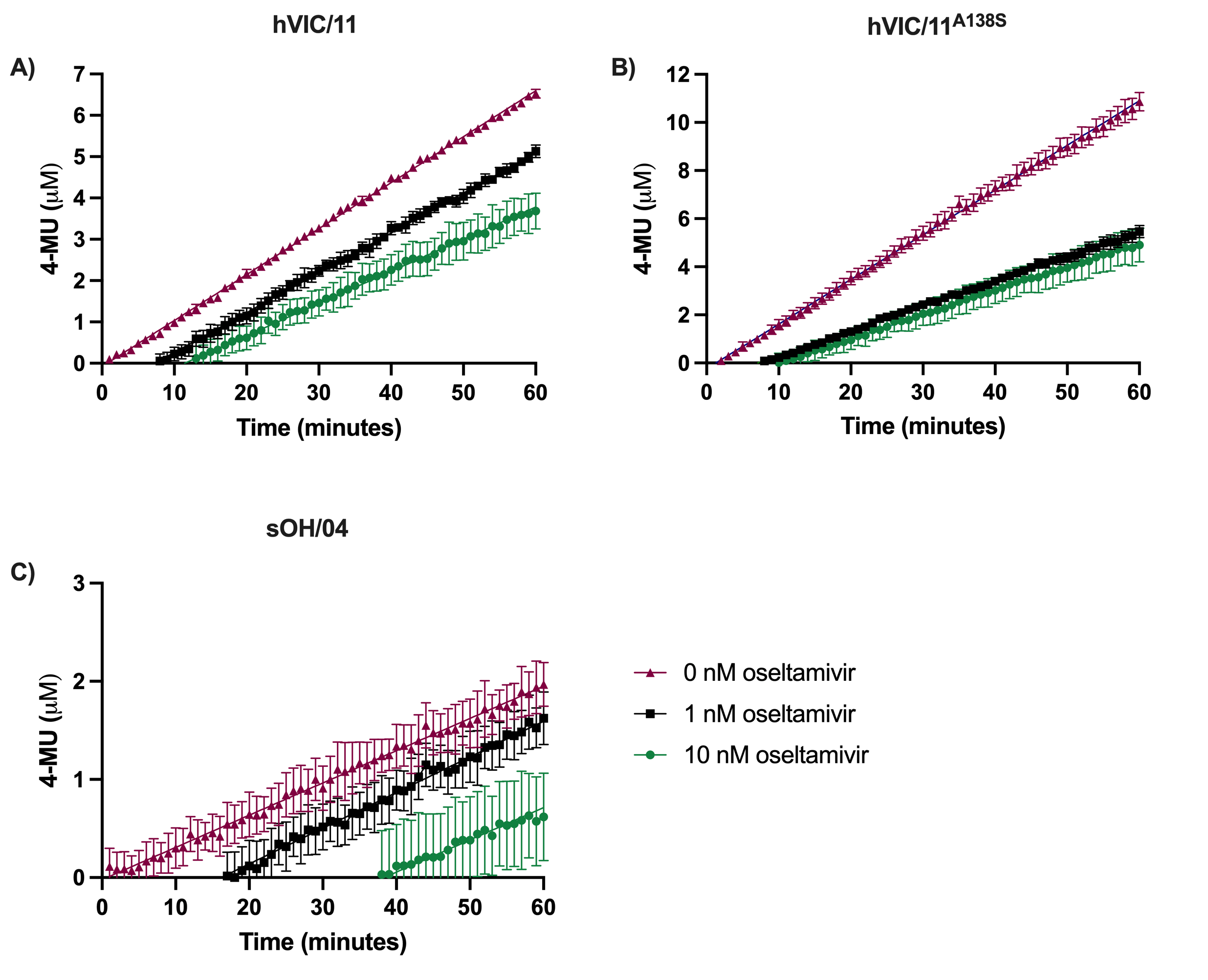

Supplement: S4 Fig — Neuraminidase activity of hVIC/11 (A), hVIC/11A138S (B), and sOH/04 (C) in presence of 0, 1, and 10 nM oseltamivir normalized to 10,000 PFU showing a dose-dependent decrease in NA activity using MUNANA as substrate and supporting the validity of the assay. (TIFF) [file ppat.1012026.s004.tiff]

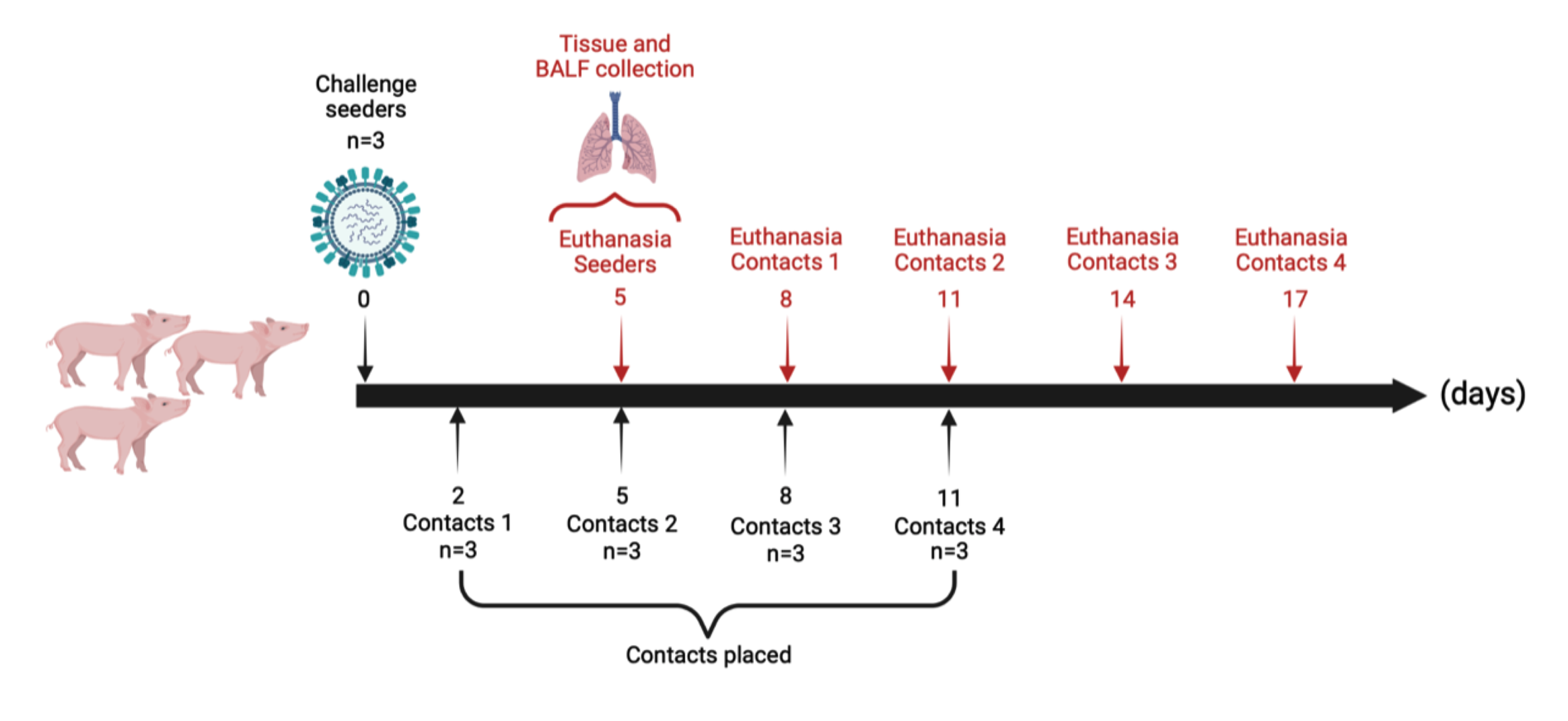

Supplement: S5 Fig — Pigs were inoculated with 3 x 10^6^ TCID50/pig of sOH/04, or hVIC/11, or hVIC/11A138S. At 2 days post-infection (dpi), naïve pigs were placed in contact with inoculated pigs. After 3 days (5dpi), new contacts were introduced after removal of inoculated pigs, and this cycle was repeated for a total of 4 contacts. Pigs were euthanized at 5 dpi/6 days post contact, and bronchoalveolar lavage fluid and lung tissues were collected from seeders pigs. This illustration was created with BioRender.com. (TIFF) [file ppat.1012026.s005.tiff]

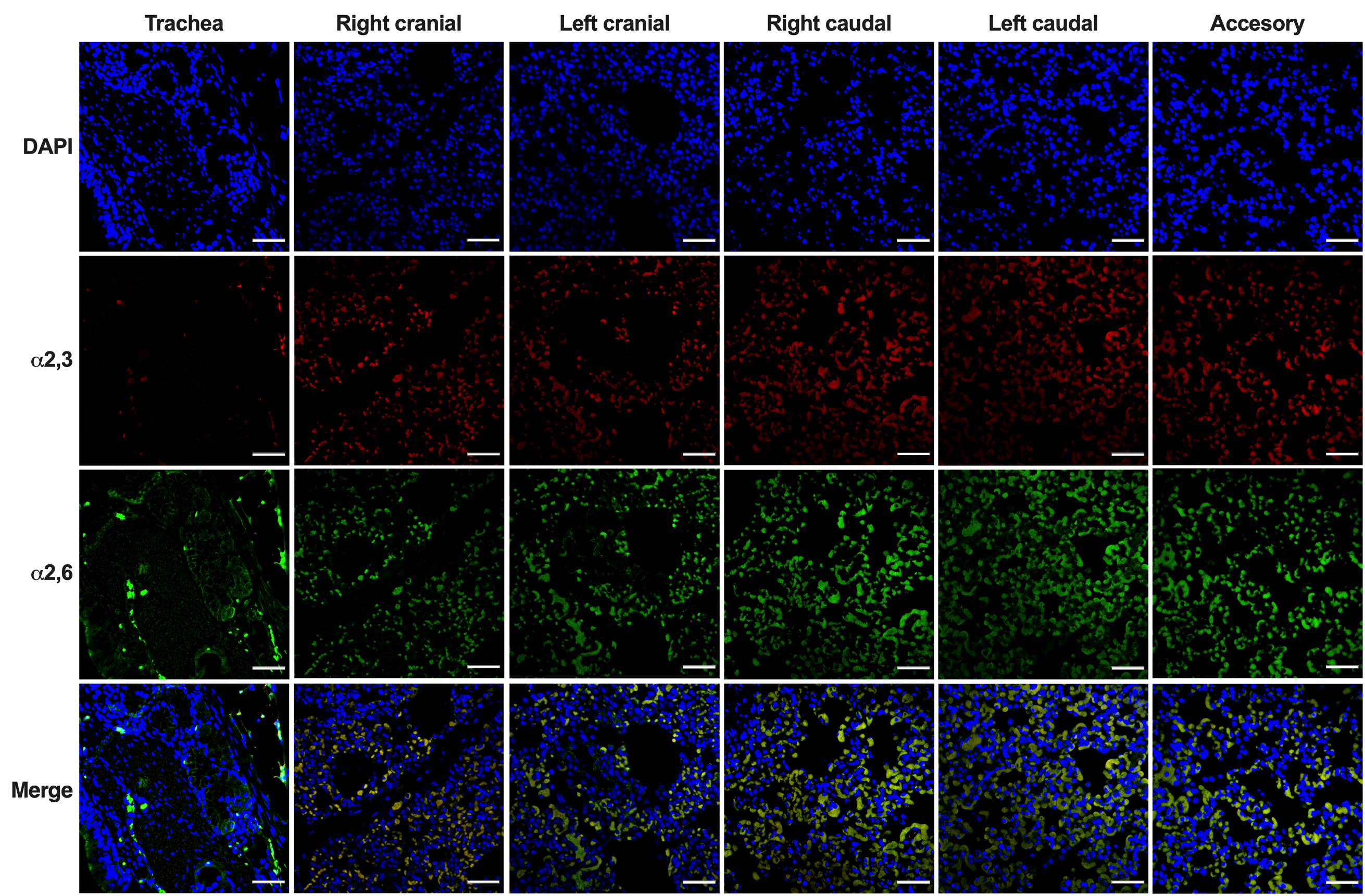

Supplement: S6 Fig — Representative confocal images showing α2,3 (MAL II, red) and α2,6 (SNA, green) receptors distribution in the upper and lower respiratory tract. α2,6 receptors are predominant in the trachea while in the right cranial, left cranial, right caudal, left caudal, and accessory lobes both receptors are evenly distributed. Cells nuclei was stained with DAPI (blue). The scale bar represents 50 μm. (TIFF) [file ppat.1012026.s006.tiff]

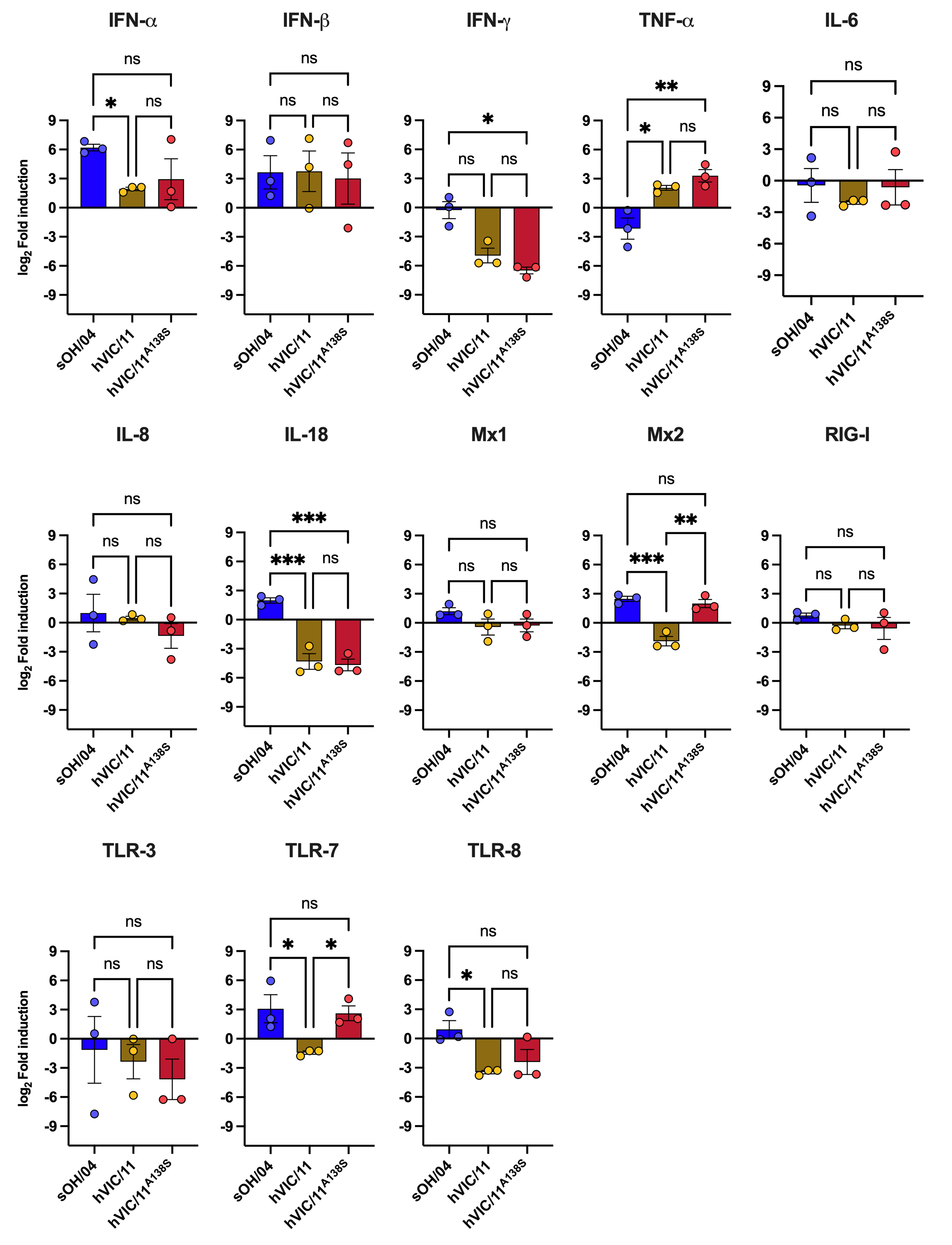

Supplement: S7 Fig — RNA was normalized to 1μg and gene expression was assessed by qPCR and normalized to RLP-19 expression in. Values are shown as log2 fold induction of the mean between the seeder pigs (n = 3) of each group at 5dpi. Fold induction of each group was normalized to the non-infected negative control group. Values represent the mean ± SEM. Statistical analysis was performed by two-way ANOVA. *p<0.05, **p<0.005, ***p<0.0005. (TIFF) [file ppat.1012026.s007.tiff]

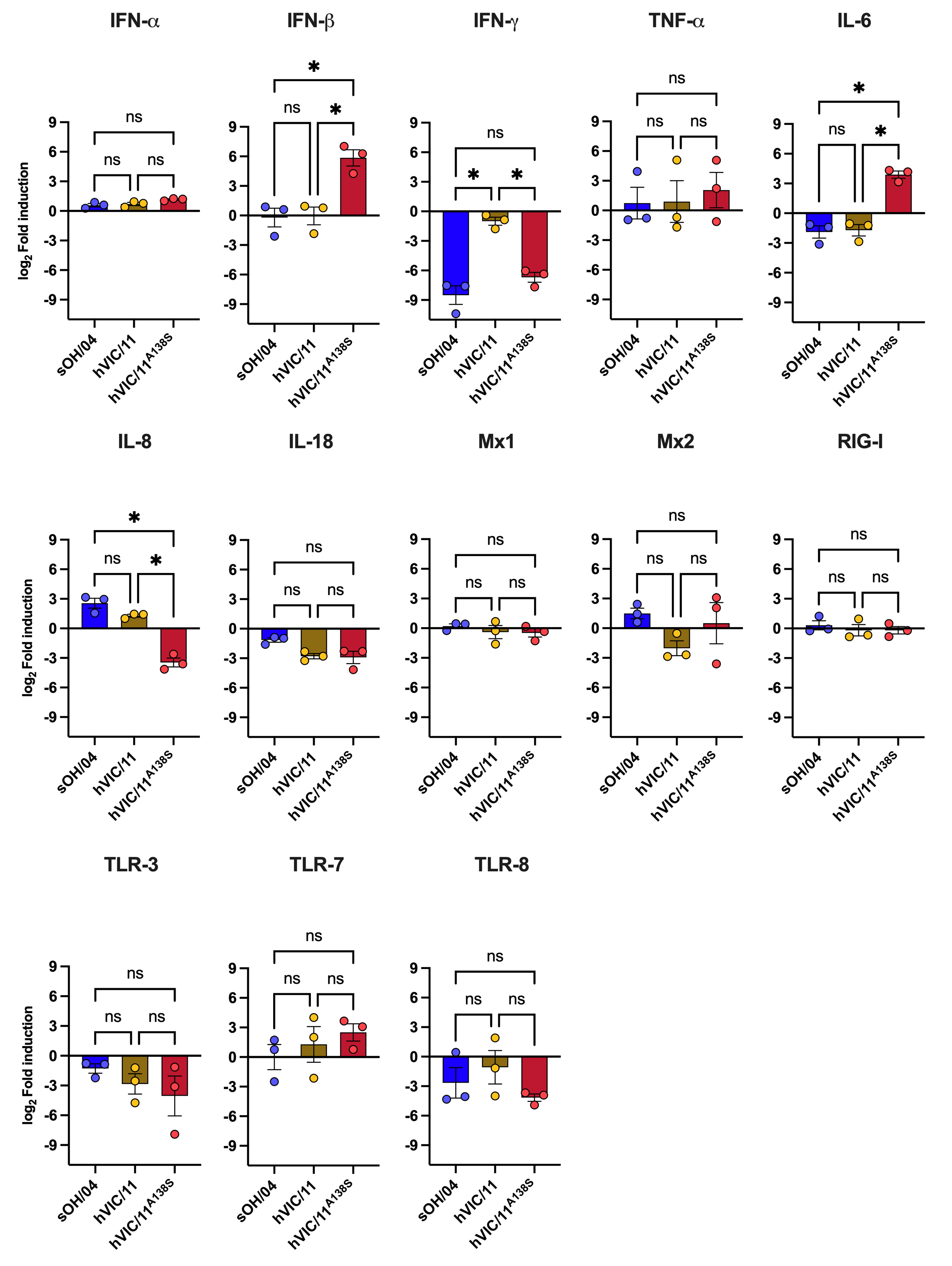

Supplement: S8 Fig — RNA was normalized to 1μg and gene expression was assessed by qPCR and normalized to RLP-19 expression in. Values are shown as log2 fold induction of the mean between the seeder pigs (n = 3) of each group at 5dpi. Fold induction of each group was normalized to the non-infected negative control group. Values represent the mean ± SEM. Statistical analysis was performed by two-way ANOVA. *p<0.05, **p<0.005, ***p<0.0005. (TIFF) [file ppat.1012026.s008.tiff]

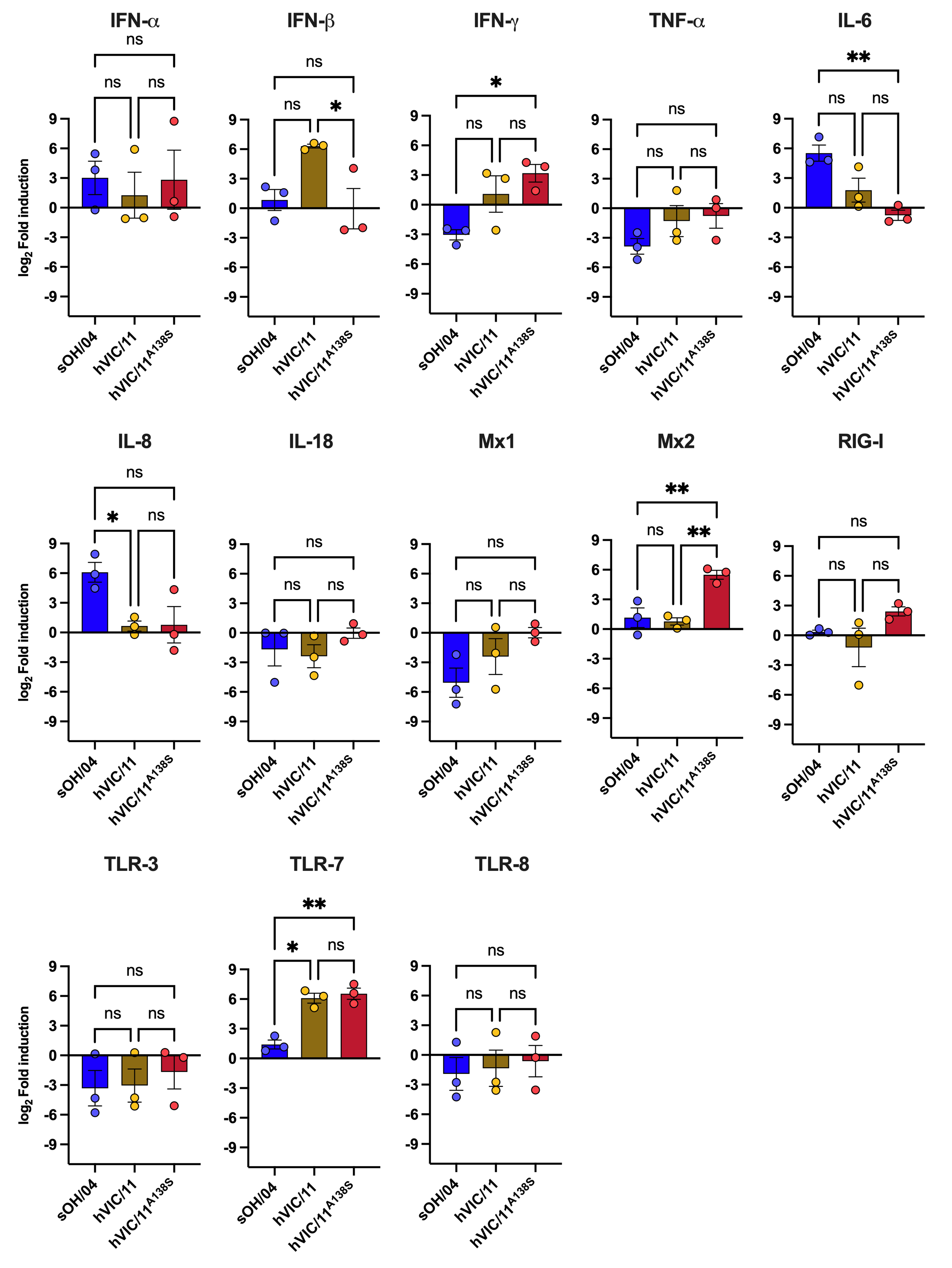

Supplement: S9 Fig — RNA was normalized to 1μg and gene expression was assessed by qPCR and normalized to RLP-19 expression in. Values are shown as log2 fold induction of the mean between the seeder pigs (n = 3) of each group at 5dpi. Fold induction of each group was normalized to the non-infected negative control group. Values represent the mean ± SEM. Statistical analysis was performed by two-way ANOVA. *p<0.05, **p<0.005, ***p<0.0005. (TIFF) [file ppat.1012026.s009.tiff]

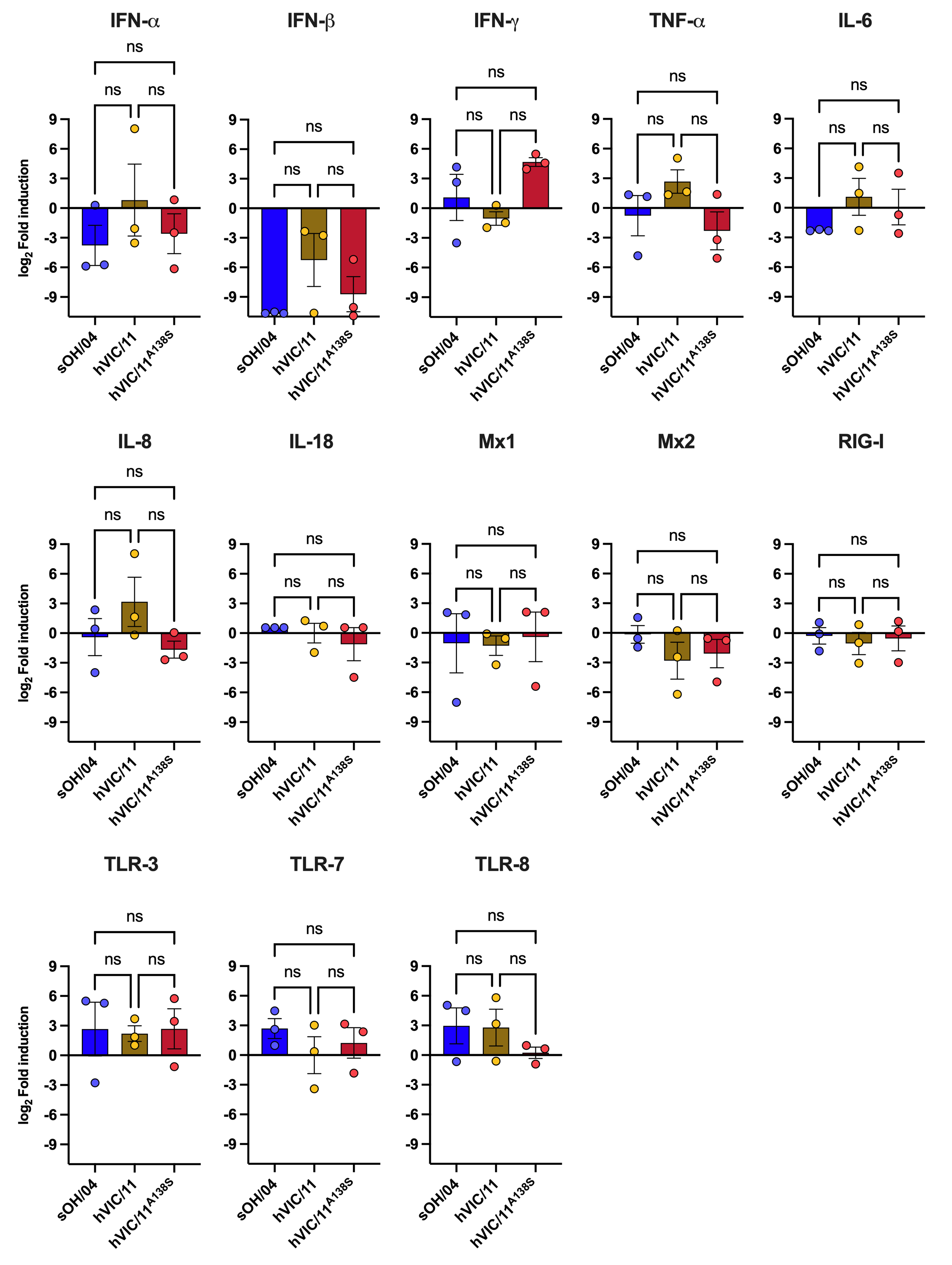

Supplement: S10 Fig — RNA was normalized to 1μg and gene expression was assessed by qPCR and normalized to RLP-19 expression in. Values are shown as log2 fold induction of the mean between the seeder pigs (n = 3) of each group at 5dpi. Fold induction of each group was normalized to the non-infected negative control group. Values represent the mean ± SEM. Statistical analysis was performed by two-way ANOVA. *p<0.05, **p<0.005, ***p<0.0005. (TIFF) [file ppat.1012026.s010.tiff]

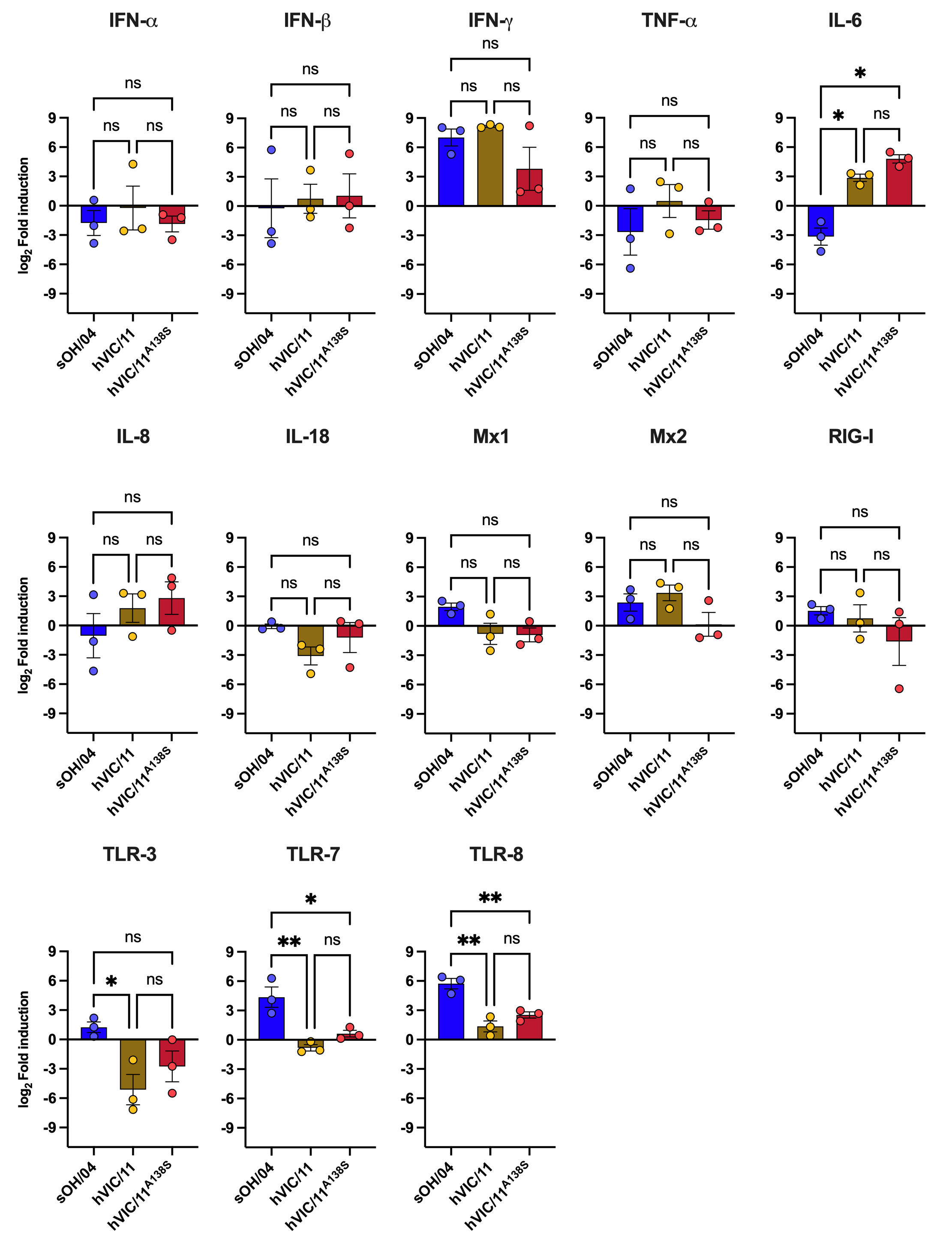

Supplement: S11 Fig — RNA was normalized to 1μg and gene expression was assessed by qPCR and normalized to RLP-19 expression in. Values are shown as log2 fold induction of the mean between the seeder pigs (n = 3) of each group at 5dpi. Fold induction of each group was normalized to the non-infected negative control group. Values represent the mean ± SEM. Statistical analysis was performed by two-way ANOVA. *p<0.05, **p<0.005, ***p<0.0005. (TIFF) [file ppat.1012026.s011.tiff]

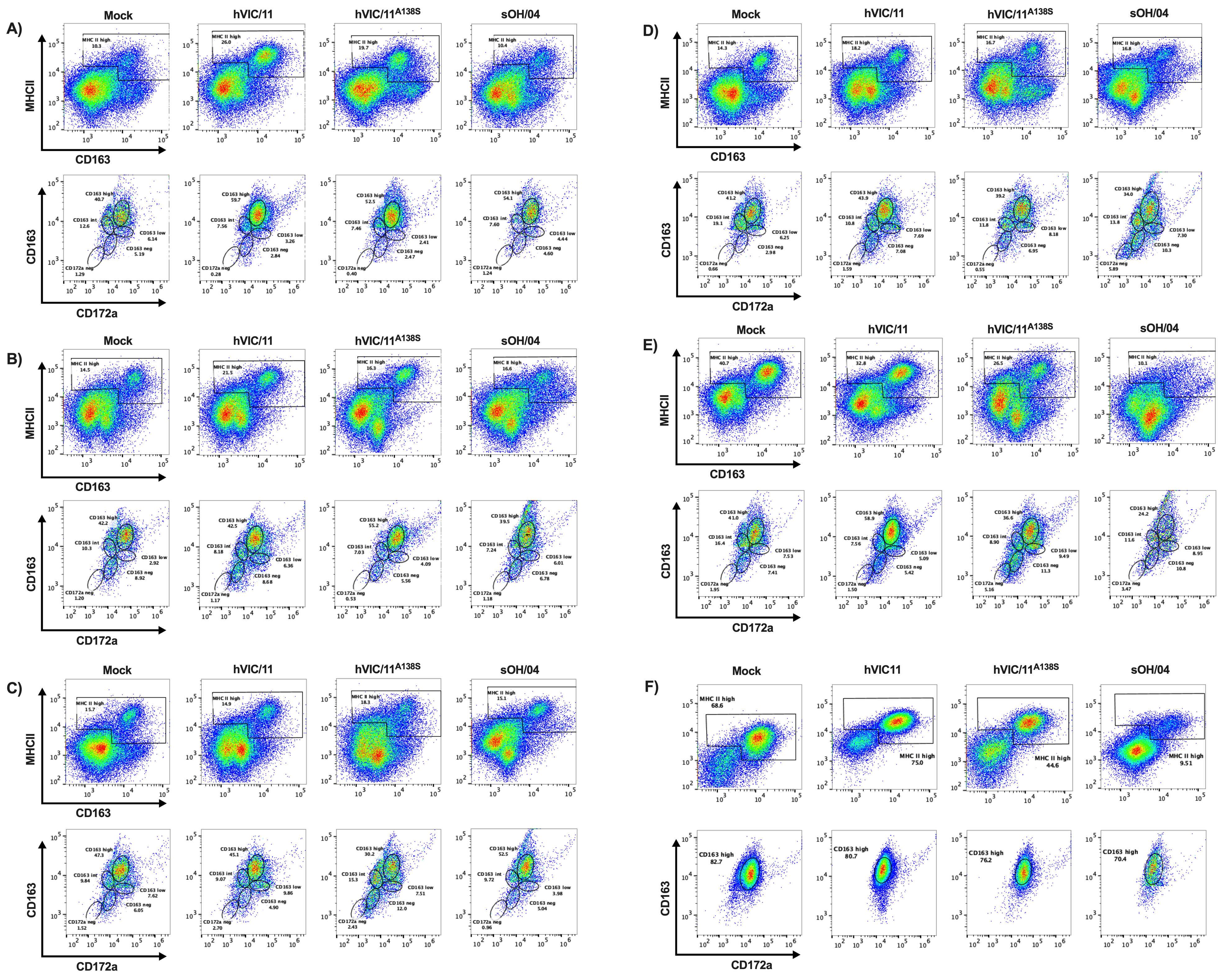

Supplement: S12 Fig — Representative flow cytometry results showing APC (top panels) and cDC1, cDC2, moDC, moMϕ, and PiAMs (bottom panels) in right cranial (A), left cranial (B), right caudal (C), left caudal (D), and accessory lobes (E) from mock-, hVIC/11-, hVIC/11A138S-, and sOH/04-infected pigs. The gating strategy performed in tissue samples was also used in BALF (F) samples and only one population gated from MHCIIhigh CD163pos cells appeared. (TIFF) [file ppat.1012026.s012.tiff]
